# Enhancing electrocatalytic hydrogen evolution *via* engineering unsaturated electronic structures in MoS_2_

**DOI:** 10.1039/d4sc07309f

**Published:** 2024-12-18

**Authors:** Qingqing Zhou, Hao Hu, Zhijie Chen, Xiao Ren, Ding Ma

**Affiliations:** a Beijing National Laboratory for Molecular Sciences, College of Chemistry and Molecular Engineering, Peking University Beijing 100871 China dma@pku.edu.cn; b College of Environment, Zhejiang University of Technology Hangzhou 310012 PR China; c School of Civil and Environmental Engineering, The University of New South Wales Sydney NSW 2052 Australia

## Abstract

The search for efficient, earth-abundant electrocatalysts for the hydrogen evolution reaction (HER) has identified unsaturated molybdenum disulfide (MoS_2_) as a leading candidate. This review synthesises recent advancements in the engineering of MoS_2_ to enhance its electrocatalytic properties. It focuses on strategies for designing an unsaturated electronic structure on metal catalytic centers and their role in boosting the efficiency of the hydrogen evolution reaction (HER). It also considers how to optimize the electronic structures of unsaturated MoS_2_ for enhanced catalytic performance. This review commences with an examination of the fundamental crystal structure of MoS_2_; it elucidates the classical unsaturated electron configurations and the intrinsic factors that contribute to such electronic structures. Furthermore, it introduces popular strategies for constructing unsaturated electronic structures at the atomic level, such as nanostructure engineering, surface chemical modification and interlayer coupling engineering. It also discusses the challenges and future research directions in the study of MoS_2_ electronic structures, with the aim of broadening their application in sustainable hydrogen production.

## Abstract 2

Focusing on the efficient electronic structures in MoS_2_. This review critically examines the origins of unsaturated electronic structures, strategies for their construction, and future directions for advancing this promising field.

## Graphical abstract

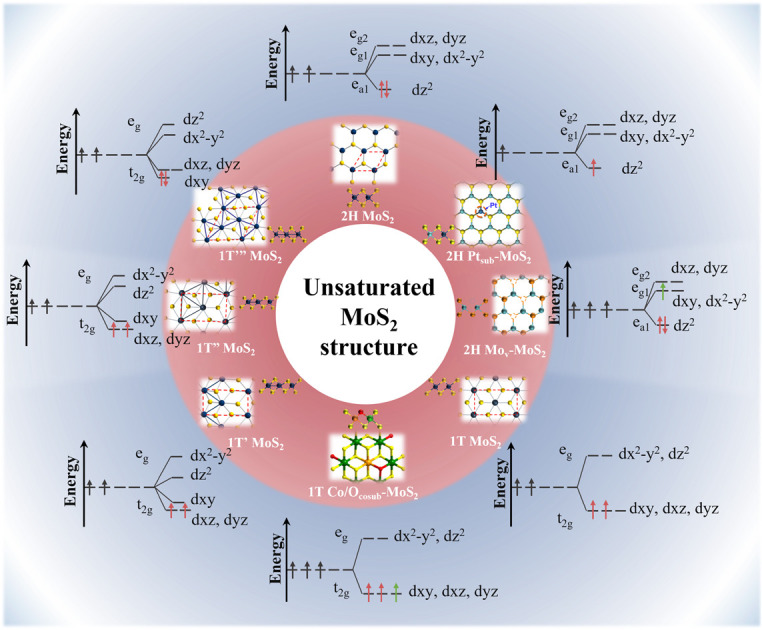

## 1 Introduction

As global demand for clean energy continues to rise, the development of efficient hydrogen production has emerged as a critical focus. Water electrolysis is a particularly promising approach due to its efficiency and environmentally friendly nature.^1–3^ However, its implementation depends heavily on the development of high-performance and durable catalysts for the hydrogen evolution reaction (HER).^4,5^ Traditionally, precious metals like platinum (Pt) have been the catalysts of choice due to their exceptional HER activity, which is attributed to their moderate binding interaction with key adsorbates, such as intermediate hydrogen species (H*).^6,7^ However, the scarcity and high cost of Pt and other noble metals have driven increasing interest in the search for earth-abundant alternatives.^8–10^ This shift is crucial to making hydrogen production more economically viable and scalable for widespread clean energy applications.

Using density functional theory (DFT) calculations, Hinnemann and colleagues identified earth-abundant materials with catalytic properties similar to platinum (*i.e.*, near-zero free energy for H* binding) for HER.^11^ Among these candidates, molybdenum disulfide (MoS_2_) was predicted as a promising HER catalyst, despite practical bulk MoS_2_ being regarded as a poor electrocatalyst.^12,13^ This apparent contradiction was later clarified by Jaramillo and Ib Chorkendorff, who demonstrated that hydrogen evolution predominantly occurs at the edge sites of layered MoS_2_, where unsaturated electronic structures are present.^14^ In contrast, the basal plane of MoS_2_, with its fully saturated electronic configuration, contributes negligibly to HER due to a lack of active sites.^15,16^ These findings underscore the significance of engineering unsaturated electronic structure of MoS_2_, supporting Norskov's perspective that the electrocatalytic performance of a material is fundamentally determined by its electronic structure.

Beyond exploring the atomic-level structure–activity relationship, numerous studies have focused on maximizing the intrinsic catalytical performance of MoS_2_ while developing additional electric properties to contribute HER progress. For example, Jin and Cui pioneered the use of Li^+^ intercalation to successfully tune the electronic states of MoS_2_, transforming semiconducting 2H-MoS_2_ into metallic 1T-MoS_2_. Their research demonstrated that Li^+^ intercalation introduces single electrons into the Mo d-orbitals, achieving a new stable state through d-orbital re-splitting. This transformation significantly enhances hydrogen species activation and improves charge transfer kinetics.^12,17^ In a complementary study, Tsai and colleagues introduced sulfur defects in the basal plane of MoS_2_, generating high-energy dangling bonds that increased the mass activity of the catalyst. Their findings suggest that thermodynamically unstable, coordinatively unsaturated sites are more prone to adsorbing H* intermediates, playing a key role in the HER process.^18^ Beyond structural modifications to the 2H phase of MoS_2_, researchers have explored the introduction of various metal and non-metal dopants to fine-tune the local coordination environment, electron density, and energy level structure of the catalytic centers. For instance, DFT calculations by Cui predicted how different transition metal dopants (*e.g.*, Co, Fe, Ni, and Cu) impact the HER performance of MoS_2_.^19^ These strategies demonstrate that engineering unsaturated electron configurations in MoS_2_ can significantly enhance its electrochemical properties, paving the way for more efficient HER catalysts.

While it has vaguely perceived that unsaturated sites in MoS_2_ are somehow linked to its electrocatalytic performance, the precise nature of this relationship remains unclear. To address this, researchers have conducted extensive studies to establish design principles for MoS_2_ catalysts with high HER activity, particularly focusing on the interaction between intermediate hydrogen species and catalytic centers.^20–23^ Previous reviews have categorized modification methods for MoS_2_ catalysts, aiming for advanced technologies that offer high catalytic activity, stability, preparation efficiency, and *etc.* In contrast, our review provides a systematic summary of the unsaturated electronic structures in modified MoS_2_ catalysts from the perspective of band structure and electronic orbital arrangement, which are decisive factors in determining electrocatalytic performance. Additionally, we provide practical guidance for how to construct active catalytic centers with idea unsaturated electronic structures: (1) through nanostructure engineering, single-layer or multi-exposed-edge MoS_2_ materials are synthesized without altering their chemical composition. Additionally, stress regulation is employed to introduce or relieve lattice strain, facilitating the formation of active defects in the distorted structure. (2) Surface chemical modification strategies-such as creating vacancy defects, heteroatom substitution, heterogeneous doping, and incorporating heterogeneous crystal domains-are used to modify the local chemical environment of the catalytic centers. These methods regulate the chemical properties of MoS_2_ at the micro-regional level, thereby enhancing its HER performance. (3) Semiconductor/metal contact effects have emerged as a promising approach for constructing robust and highly efficient catalytic electrodes. By modulating the van der Waals gap between MoS_2_ catalytic layers and metal supports, the orbital overlap and hybridization at the interface can regulate the in-plane electronic structure of MoS_2_, thereby promoting the exposure of active centers and enhancing HER efficiency. Finally, we discuss the challenges and future research directions in optimizing the electronic structures of MoS_2_ catalysts, with the aim of promoting their broader application in sustainable hydrogen production.

## 2 Fundamentals of MoS_2_ electrocatalyst for HER

### 2.1 HER mechanism

HER is a two-electron/proton coupling process that occurs on the cathode surface during water electrolysis, with the reaction mechanism dependent on the pH of the solution.^24,25^ As outlined in Table 1, the HER involves two main steps: adsorption (Volmer) and desorption (Heyrovsky/Tafel). First, the Volmer step initiates the reaction, where hydrogen ions (in acidic environments) or water molecules (in alkaline environments) are electrochemically adsorbed onto the catalyst surface, forming an intermediate hydrogen species (H_ads_). The Heyrovsky and Tafel steps describe two desorption pathways for H_ads_. The Heyrovsky step, predominant at low H_ads_ surface coverage, involves H_ads_ reacting with another hydrogen ion or water molecule to form H_2_. The Tafel step, occurring at high H_ads_ surface coverage, involves the combination of two H_ads_ to produce H_2_.^26^ It is clear that an effective HER catalyst is essential for optimizing these adsorption and activation pathways.

**Table 1:** HER pathways in acid and alkaline solution

	Acid solution	Alkaline solution
Volmer step	M + H^+^ + e^−^ → H_ads_	M + H_2_O + e^−^ → H_ads_ + 2OH^−^
Heyrovsky step	M–H_ads_ + H^+^ + e^−^ → H_2_	M–H_ads_ + H_2_O + e^−^ → H_2_ + OH^−^
Tafel step	2M–H_ads_ → H_2_ + 2M	2M–H_ads_ → H_2_ + 2M
Overall reaction	2H^+^ + 2e^−^ → H_2_	2H_2_O + 2e^−^ → H_2_ + 2OH^−^

### 2.2 Basics of MoS_2_ crystal

#### 2.2.1 Phase and stacking of MoS_2_ crystal

**Fig. 1:**
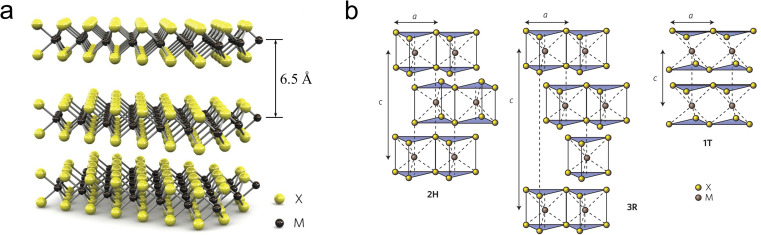
(a) Schematic diagram of the MoS_2_ crystal structure, (b) schematic diagram of the structures of 2H phase MoS_2_ (hexagonal symmetry, two layers per repeating unit, trigonal prism coordination), 3R phase MoS_2_ (rhombohedral symmetry, three layers per repeating unit, trigonal prism coordination) and 1T phase MoS_2_ (tetragonal symmetry, one layer per repeating unit, octahedral coordination), the stacking index *c* represents the number of layers in each stacking sequence, and the interlayer spacing is about 6.5 Å.

A monolayer of MoS_2_ has a graphene-like 2D structure, where a layer of molybdenum (Mo) atoms is sandwiched between two layers of sulfur (S) atoms, forming an S–Mo–S stacking arrangement.^27,28^ The layers of MoS_2_ are held together by weak van der Waals (vdW) interactions, with a repeat distance of 6.5 Å between them, while the in-plane structure is stabilized by strong Mo–S covalent bonds (Fig. 1a).^29^

As depicted in Fig. 1b, depending on the specific coordination of Mo atoms, MoS_2_ cell can exist in either a trigonal prismatic or octahedral configuration.^30^ Based on the phase structure and stacking sequence, MoS_2_ has three standard crystal structures: 2H phase (H stands for hexagonal) with two S–Mo–S molecular units stacking in an AB manner along the vertical (*c*-axis) direction; 3R (R stands for rhombohedral) with three S–Mo–S units stacked in an ABC manner along the *c*-axis; 1T phase (T stands for trigonal) with a monolayer S–Mo–S molecular per unit cell.^31,32^ The energy level splitting, electron filling, and spin states of the Mo d-orbitals vary with the coordination structure, leading to differences in electrical properties, magnetic properties, and thermodynamic stability.^33,34^ Among these, 2H-MoS_2_ is the most stable, while the 1T and 3R phases exhibit metastable behavior.^35^

#### 2.2.2 Band structure &electronic properties of MoS_2_

As a typical transition metal dichalcogenides (TMDs) material, the electronic properties of MoS_2_ are determined by the interactions between the p, s, and d orbitals of each atom near the Fermi level. Typically, the contribution of the S s orbitals is neglected because of their minimal perturbation on the band structure. In terms of energy, the orbital energy of S atoms is significantly lower than that of the Mo d orbitals. Consequently, the electronic structure and properties of MoS_2_ are primarily influenced by the coordination environment and the d orbital energy levels of the Mo atoms.^34^ The unit cell of MoS_2_ contains six p orbitals (two S atoms, each contributing three sub-bands), which can accommodate up to 12 electrons. The S atoms contribute eight electrons, while the remaining four electrons are provided by the Mo atoms. Consequently, only two electrons are available for allocation to the Mo d orbitals (as the Mo atom, with six valence electrons, gives four to the S atoms).

**Fig. 2:**
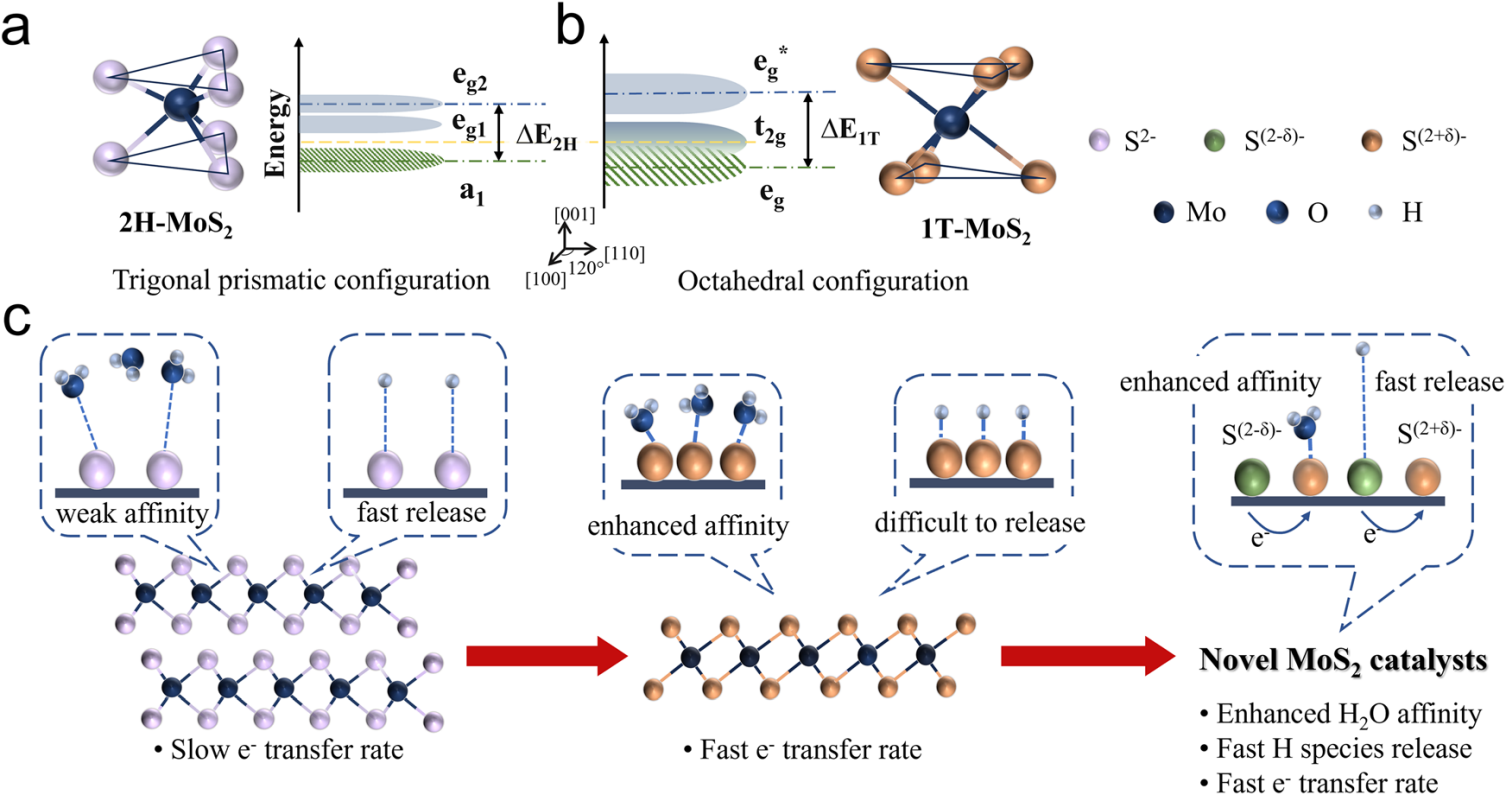
The unit cell structure, band structure and Fermi level position of (a) 2H phase MoS_2_ and (b) 1T phase MoS_2_. (c) HER catalytic behavior on 2H phase MoS_2_, 1T phase MoS_2_, and the principle to modify MoS_2_.

Due to the varying orientations and shapes of the d orbitals in molybdenum ions, the d orbitals of the central metal atom experience electrostatic interactions with ligands that differ in both direction and energy depending on the crystal field environment. To clarify, 1T-phase MoS_2_ adopts an octahedral molecular configuration with an *O*_h_ point group, where six S ligands are positioned at the vertices of a symmetric octahedron surrounding the Mo atom. The electron clouds of the d_*z*^2^_ and d_*x*^2^−*y*^2^_ orbitals are located on the connecting line of Mo–S and directly face the negatively charged ligands, resulting in stronger repulsive interactions and higher energy levels, e_g_ band.^36,37^ On the contrary, the electron clouds of the d_*xy*_, d_*yz*_, and d_*xz*_ orbitals with spatial deviation from the Mo–S axis and avoid the ligands, leading to weaker repulsive interactions and lower energy t_2g_ band (Fig. 2b). These degenerate orbitals remain non-bonding, allowing for quasi-continuous occupation by fermions, thereby giving MoS_2_ metallic properties. However, for nonlinear molecules, if their ground electronic state is orbitally degenerate, the molecule becomes unstable. In such cases, the Jahn–Teller distortion effect occurs to relieve the degeneracy.

The molecular configuration of 2H-MoS_2_ is such a distorted octahedron. By rotating one triangular face by 60° relative to the opposite face, a trigonal prismatic molecular configuration, point group *D*_3h_, is obtained. In this trigonal prismatic configuration, due to the lack of symmetrical alignment between the S ligands and the Mo 4d orbitals, the d orbitals tend to overlap “sideways” resulting in repulsion from all ligands. This causes the molecular orbitals to split into bonding and antibonding bands, creating an empty band gap. In this case, the d orbitals split into three bands: e_g2_, e_g1_, and a_1_, corresponding to the d_*xz*_, d_*yz*_; d_*xy*_, d_*x*^2^−*y*^2^_; and d_*z*^2^_, respectively (Fig. 2a).

As discussed above, the 2H and 1T crystalline phases of MoS_2_, the two most widely applied phases, exhibit fundamentally distinct solid-state physical and electronic properties, resulting in notable differences in HER catalytic performance. Regarding the HER process (Fig. 2c), the semiconducting 2H phase is characterised by a significant band gap and limited electron mobility. Additionally, its strong bonding within the basal plane provides excellent thermodynamic stability but renders it catalytically inert. Consequently, the HER activity of 2H-MoS_2_ primarily originates from its exposed edge sites, where unsaturated electronic states facilitate the adsorption and conversion of intermediates. This highlights edge engineering as a critical strategy for enhancing the catalytic performance of the 2H phase. In contrast, the metallic 1T phase offers excellent electronic conductivity, enabling rapid electron transfer during the HER process. Furthermore, the high density of states near the Fermi level strengthens interactions between 1T-MoS_2_ and water molecules, promoting efficient adsorption and activation of intermediates. However, the 1T phase suffers from structural instability, often requiring stabilization strategies such as intercalation or heteroatom doping to maintain its catalytic performance under operational conditions.

### 2.3 Relationship between electron configuration of MoS_2_ and its electrocatalytic activity

**Fig. 3:**
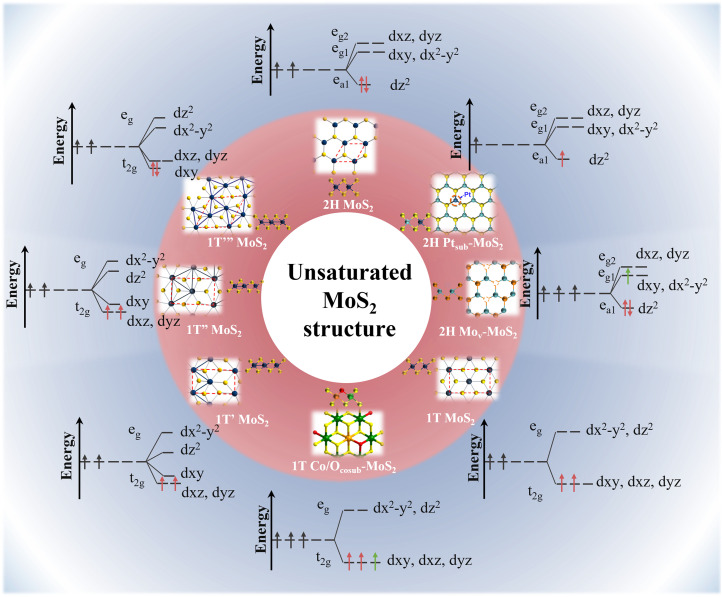
Orbital electron unsaturation strategy for MoS_2_ electrocatalysts based on crystal field theory.

Generally, the basal plane of MoS_2_ exhibits limited catalytic activity due to its saturated electronic configuration.^38,39^ However, modifying the coordination environment of the Mo centers and tuning the energy levels of their d orbitals offer significant opportunities for creating high-activity, unsaturated MoS_2_ configurations, thereby enhancing its catalytic potential for HER.^40,41^ Numerous efforts have been made in this direction, as presented in as presented in Fig. 3 and Table 2. To clarify, in the ground-state 2H-MoS_2_ with a trigonal prismatic structure, the crystal field splitting energy is greater than the electron pairing energy. Following the Aufbau principle, electrons first occupy the low-energy d_*z*^2^_ orbital with two electrons of opposite spins, resulting in diamagnetism. Interestingly, when the host Mo atom is substituted by noble metals such as Pt and Rh, electrons are lost from the d_*z*^2^_ orbital due to the higher electronegativity of these metals.^42,43^ Consequently, this results in a low-spin unsaturated state that exhibits metallic-like properties.

**Table 2:** Electronic band structure and characteristics of unsaturated MoS_2_

Catalysts	Phase	Modification approach	Description of band structure and electrons state	Conductivity	Ref.
Pristine MoS_2_	2H	—	Three bands: a_1_ (d_*z*^2^_), e_g1_ (d_*xy*_, d_*x*^2^−*y*^2^_), and e_g2_ (d_*yz*_, d_*xz*_)	Semi-conductor	34
			Electron state: paired electrons fully occupy the lowest a_1_ band		
Pt-MoS_2_	2H	Mo loses electrons through substitution with highly electronegative noble metals	Three bands: a_1_ (d_*z*^2^_), e_g1_ (d_*xy*_, d_*x*^2^−*y*^2^_), and e_g2_ (d_*yz*_, d_*xz*_)	Metallic-like	42
			Electron state: single-spin electron half-fill the lowest a_1_ band		
FD-MoS_2_	2H	Mo losing electrons by making Frenkel disorder	Three bands: a_1_ (d_*z*^2^_), e_g1_ (d_*xy*_, d_*x*^2^−*y*^2^_), and e_g2_ (d_*yz*_, d_*xz*_)	Metallic-like	42
			Electron state: single-spin electron half-fill the lowest a_1_ band		
V_s_-MoS_2_	2H	Adding electrons by making S vacancy	Three bands: a_1_ (d_*z*^2^_), e_g1_ (d_*xy*_, d_*x*^2^−*y*^2^_), and e_g2_ (d_*yz*_, d_*xz*_)	Metallic-like	43
			Electron state: paired electrons fully occupy the lowest a_1_ band, and single-spin electron incompletely occupy the higher e_g1_ band		
1T-MoS_2_	1T	—	Two bands: t_2g_ (d_*xy*_, d_*yz*_, d_*xz*_) and e_g_ (d_*x*^2^−*y*^2^_, d_*z*^2^_)	Metallic	34
			Electron state: two parallel-spin electrons incompletely occupy the degenerate t_2g_ band		
CoO@1T-MoS_2_	1T	Adding electrons by co-substituting Mo and S with Co and O atoms	Two bands: t_2g_ (d_*xy*_, d_*yz*_, d_*xz*_) and e_g_ (d_*x*^2^−*y*^2^_, d_*z*^2^_)	Metallic	44
			Electron state: three parallel-spin electrons half-fill the degenerate t_2g_ band		
1T′-MoS_2_	Stretch 1T phase	Re-split band structure by intercalating alkine ions	Two major bands: t_2g_ (d_*xy*_, d_*yz*_, d_*xz*_) and e_g_ (d_*x*^2^−*y*^2^_, d_*z*^2^_)	Metallic-like	45
			Electron state: two parallel-spin electrons half-fill the lower t_2g_ band		
1T′′-MoS_2_	Stretch 1T phase	Re-split band structure by chemical exfoliation	Two major bands: t_2g_ (d_*xy*_, d_*yz*_, d_*xz*_) and e_g_ (d_*x*^2^−*y*^2^_, d_*z*^2^_)	Metallic-like	46
			Electron state: two parallel-spin electrons half-fill the lower t_2g_ band		
1T′′′-MoS_2_	Compress 1T phase	Re-split band structure by de-intercalating of alkine ions	Two major bands: t_2g_ (d_*xy*_, d_*yz*_, d_*xz*_) and e_g_ (d_*x*^2^−*y*^2^_, d_*z*^2^_)	Semi-conductor	45
			Electron state: paired electrons fully occupy the lower t_2g_ band		

Conversely, donating additional electrons to the e_g_ band is more challenging due to the presence of a band gap. Creating vacancies can be a viable approach, as it destabilizes the lattice. This destabilization may trigger a transition from the 2H to the 1T phase upon electron absorption. However, the behavior of electrons differs significantly in the octahedral case. First, the crystal field splitting energy is lower than the electron pairing energy, allowing electrons to preferentially fill all orbitals of similar energy while avoiding pairing. As a result, the incomplete occupation of the t_2g_ orbitals in 1T-MoS_2_ leads to an intermediate spin state, which demonstrates moderate activity but low stability.

Doping 1T-MoS_2_ with electron-donating atoms (*e.g.*, Co, Mn, Fe) can achieve a half-filled t_2g_ band, promoting a higher spin state and enhanced stability.^44,47^ Furthermore, significant distortion occurs when 1T-MoS_2_ undergoes external strain, such as compression or stretching. This strain can induce the spontaneous conversion of the 1T phase into 1T′ and 1T′′ phases, thereby lowering its energy through Jahn–Teller deformation effects.^40,46^ Typically, these distorted phases feature zigzag Mo–Mo chains and the formation of Mo–Mo bonds, such as dimerized Mo (1T′′) or trimerized Mo (1T′′′) atoms. These structures may act as key catalytic sites during the HER process, although they are sometimes overlooked in catalytic evaluation.

## 3 Nanostructure engineering

The differences in atomic arrangement and geometry contribute to the diverse physical and chemical properties of MoS_2_ catalysts. This includes variations in polymorphic structures (*e.g.*, 2H and 1T phases), as well as band structure and crystal plane exposure, which are influenced by the number of layers and the structure of MoS_2_ catalysts.^48,49^ In recent years, geometry and strain engineering of nanomaterials has attracted great attention by adjusting the properties and electrocatalytic performance of MoS_2_ by regulating the crystal atomic arrangement and deformation.^50,51^ This section outlines recent advances in the development of monolayer materials, edge dangling bond engineering, and stress regulation strategies for the design of advanced MoS_2_ catalysts.

### 3.1 Atomically thin structure

**Fig. 4:**
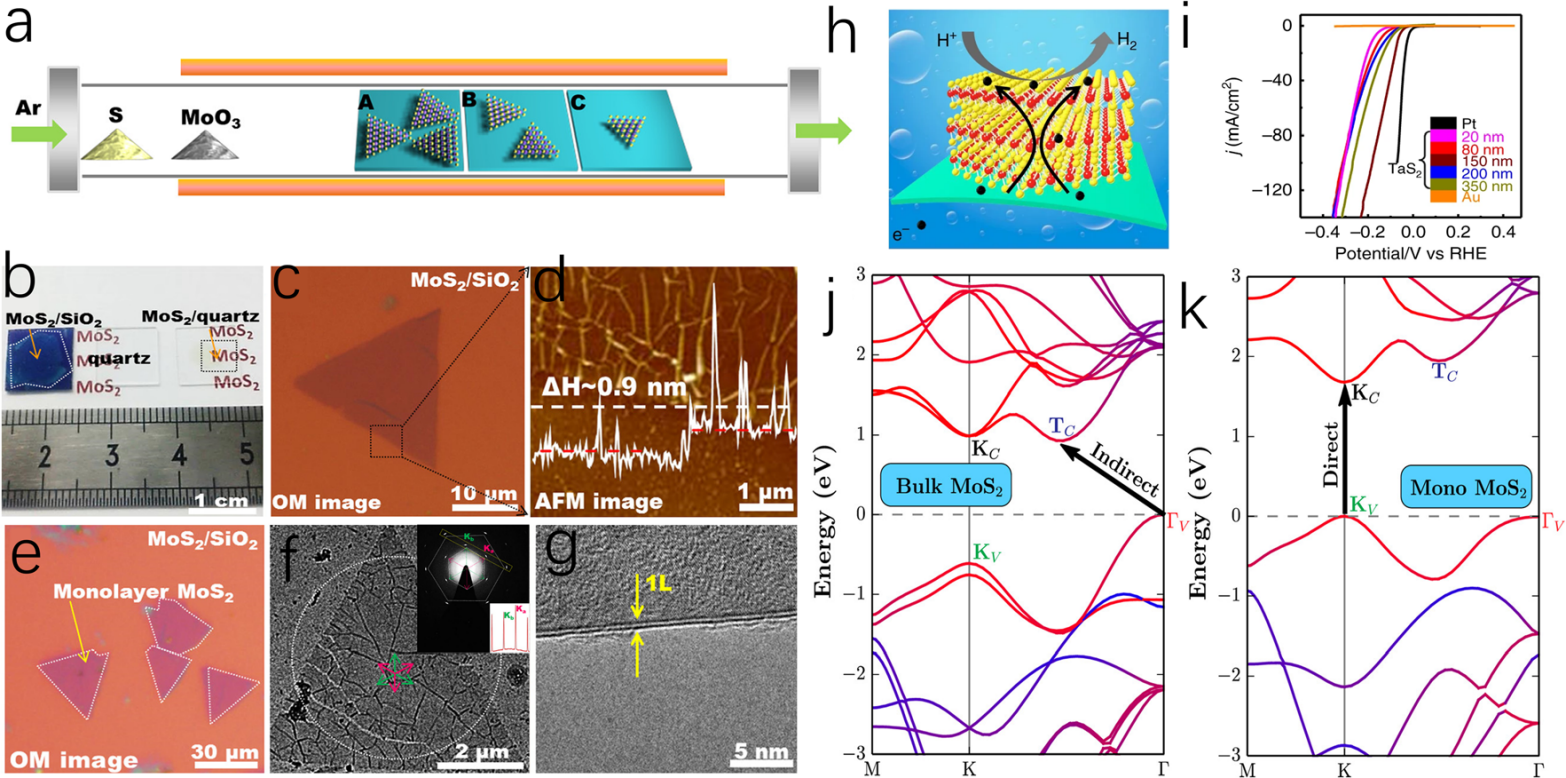
(a) Schematic representation of the dependence of coverage or flake size on different precursor-substrate distances. (b) Photographs of monolayer MoS_2_ films on SiO_2_/Si, a bare quartz substrate, and MoS_2_ on quartz. (c) A triangular MoS_2_ flake transferred onto SiO_2_/Si substrate. (d) Atomic force microscopy (AFM) image captured from the dashed box in (c) and corresponding section-view along the dashed arrow showing the monolayer feature of the flake (∼0.9 nm in apparent height). (e) Optical images of submonolayer MoS_2_ flakes after transference onto SiO_2_/Si and quartz substrates. (f) TEM image of a MoS_2_ triangle transferred on a carbon film supported on copper grids. (g) TEM image on a folded edge indicating the monolayer nature of the MoS_2_ flake. (h) Schematic illustration of the HER process of 2H-TaS_2_/Au foils. (i) Polarization curves (*iR*-corrected) of as-grown 2H-TaS_2_ with different thicknesses, Au foil, and commercial Pt. Band structure for MoS_2_ of the bulk (j) and the monolayer (k). The band structure has been projected onto constituting atomic species with the blue color representing S and red representing Mo. The band-edge states relevant to optical transition (*K*_V_, *K*_C_, *Γ*_V_, and *T*_C_) are labeled and the band gap transitions are indicated.

Bulk MoS_2_, held together by van der Waals (vdW) interactions, exhibits the properties of an indirect bandgap semiconductor.^52,53^ Differently, the monolayer MoS_2_ obtained by Novoselov *et al.* through mechanical exfoliation in 2005 is considered to have a direct band gap with metallic properties.^54^ Therefore, it was found that the electrical properties of MoS_2_ are highly related to the number of layers, and few-layer or monolayer MoS_2_ shows great potential in electrocatalysis due to its unique electrical properties.^55,56^ As depicted in Fig. 4a–g, to achieve controllable synthesis of monolayer MoS_2_, Zhang's group has focused on exploring the fabrication of atomically thin monolayer MoS_2_ films. These efforts include the epitaxial growth of monolayer WS_2_ on sapphire and the growth of monolayer MoS_2_ of various sizes on Au foil *via* low-pressure chemical vapor deposition (CVD), both of which can serve as outstanding electrocatalysts for HER.^57–59^ Based on this, Zhang and coauthors successfully prepared a four-layer 2H-TaS_2_ film on an Au foil, exposing edge active sites by controlling the number of layers. This approach achieved Pt-like hydrogen evolution efficiency, with a Tafel slope of 33–42 mV dec^−1^ and an exchange current density of ∼179.47 μA cm^−2^ (Fig. 4h and i).^60^ Additionally, Ye *et al.* achieved high-yield monolayer MoS_2_ films by hydrolyzing a lithium salt-containing MoS_2_ precursor, resulting in a monolayer MoS_2_ catalyst with a catalytic activity of 1563.6 μmol h^−1^.^61^

To elucidate the mechanism by which the electronic properties of MoS_2_ are regulated by the number of layers, Zunger *et al.* employed first-principles calculations and revealed that in bulk MoS_2_, the valence band maximum and conduction band minimum are located at the *T*_v_ and *T*_c_ points in momentum space, respectively.^62^ However, in monolayer MoS_2_, these points shift to the *K* point in the Brillouin zone, inducing a bandgap transition, as illustrated in Fig. 4j and k. Secondly, the monolayer structure of MoS_2_ stabilizes the edge regions by eliminating constraints from adjacent layers, which facilitates the exposure of edge sites and the introduction of structural defects, thereby accelerating the HER process. Although monolayer or few layer MoS_2_ catalysts exhibit excellent electrical properties and a high ratio of exposed active edges, the exfoliation and preparation of such MoS_2_ catalysts remain long-standing challenges, making it unsuitable for the large-scale production of cost-effective HER catalysts.

### 3.2 Morphology control

**Fig. 5:**
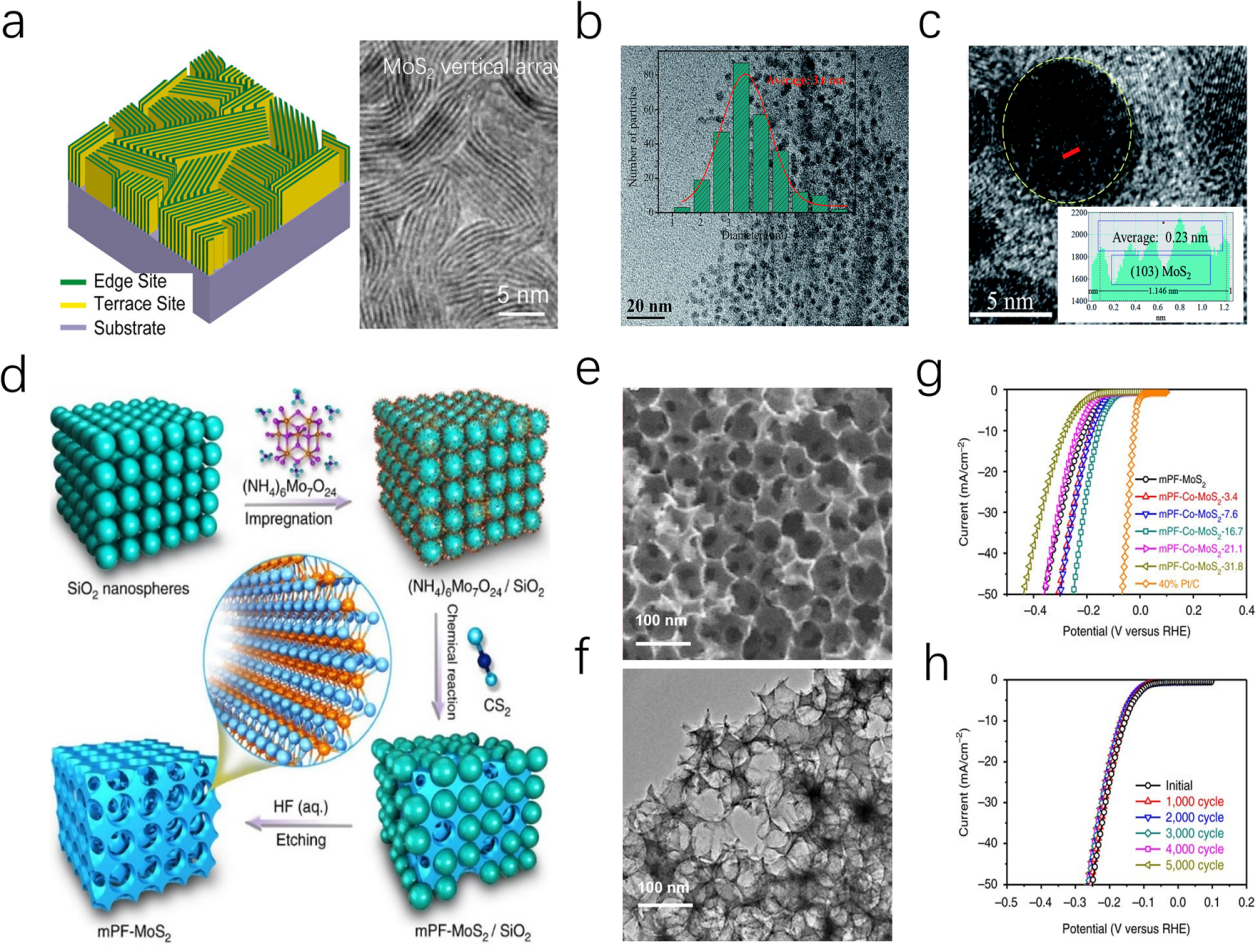
(a) (left) TEM images of MoS_2_ and MoSe_2_ films with edge-terminated structures. (right) Idealized structure of edge-terminated molybdenum chalcogenide films with the layers aligned perpendicular to the substrate. (b) TEM image of the MoS_2_ QDs, the inset displays the HRTEM image of the same. (c) The size distribution of the MoS_2_ QDs in (b). The red solid line is the simulated Gaussian curve. (d) Schematic illustration of the fabrication of mesoporous MoS_2_ foam (mPF-MoS_2_). (e and f) SEM and TEM images of mPF-MoS_2_. (g) HER polarization curves for mPF-Co-MoS_2_ with different Co doping contents in comparison with mPF-MoS_2_ and 40% Pt/C. (h) Durability measurement of mPF-Co-MoS_2_.

The highly reactive edges of MoS_2_ are typically difficult to stabilize, leading bulk MoS_2_ to terminate with smooth, inert surfaces.^63^ To overcome this limitation, researchers have exploited the anisotropy and morphological diversity of MoS_2_ nanosheets to expose and stabilize active edge sites.^64^ For instance, as depicted in Fig. 5a, Cui *et al.* grew vertically MoS_2_ nanoarray on a Mo substrate *via* a CVD method, effectively increasing the reactive area and promoting the contact between the active edges and the electrolyte.^65^ This approach enables MoS_2_ to achieve an intrinsic turnover frequency (TOF) of 0.013 s^−1^ without additional energy input, which is close to the experimental fit value (0.016 s^−1^) for pure edge sites reported by Chorkendorff *et al.*^14^ In addition, Yang and co-workers also prepared a similar Mo-based MoS_2_ electrocatalyst, reaching remarkable stability over more than 20 000 cycles in 0.5 M H_2_SO_4_.^66,67^

In addition to utilizing metal foils, MoS_2_ nanosheets can also be fabricated into mesoporous foams (mPF-MoS_2_-SiO_2_) through SiO_2_ template etching. The resulting catalyst features an average pore size of ∼30 nm, which effectively enhances the transport of H_3_O^+^ and H^+^ ions. Moreover, the directional growth of MoS_2_ nanosheets around the mesopores contributes to edge stabilization, resulting in a catalyst that demonstrates cycle durability exceeding 5000 cycles (Fig. 5d–h). Preparing MoS_2_ as highly dispersed nanoparticles is a feasible approach to obtaining a large number of accessible edge sites.^68^ Jiang and coauthors successfully synthesized highly dispersed MoS_2_ nanoparticles on reduced graphene oxide-modified carbon nanotube/polyimide films (PI/CNT-RGO-MoS_2_) through an electrochemical method. The study indicates that PI/CNT-RGO facilitates the intimate growth of MoS_2_ particles on its surface, while the coupling between RGO and MoS_2_ enhances electron transfer between the edge sites and the substrate, enabling the catalyst to exhibit a low overpotential of 90 mV at a current density of 10 mA cm^−2^ and a Tafel slope of 61 mV dec^−1^.^69^

Reducing the dimension of 2D MoS_2_ to 0D quantum dots (MoS_2_ QDs) is another advanced strategy to achieve an ultra-high specific surface area and a unique edge-defect-rich structure (Fig. 5b and c). Moreover, monolayer MoS_2_ QDs can eliminate the interlayer barrier in the vertical direction, allowing more free electrons to transfer along the MoS_2_ surfaces. Under acidic conditions, the MoS_2_ QDs catalyst developed by Ren exhibits an overpotential of 39 mV at a current density of 10 mA cm^−2^, which is 14 times lower than that of bulk MoS_2_.^70^ More interestingly, Jaramillo *et al.* reported a unique double-helical MoS_2_ structure (DG) MoS_2_ film, which exposed a greater fraction of catalytically active edge sites through high surface curvature and promoted the diffusion process of H_2_ bubbles.^63^ Various morphology designs not only increase the active specific surface area of MoS_2_ catalysts but also expand their application potential. On one hand, morphology control allows for the manipulation of the micro-interface environment of electrodes, improving mass transfer behavior of the reactants. Additionally, in Amper-scale HER applications, nanoarray morphologies are expected to facilitate efficient H_2_ bubble desorption and diffusion, addressing kinetic challenges posed by massive hydrogen accumulation.

### 3.3 Strain engineering

**Fig. 6:**
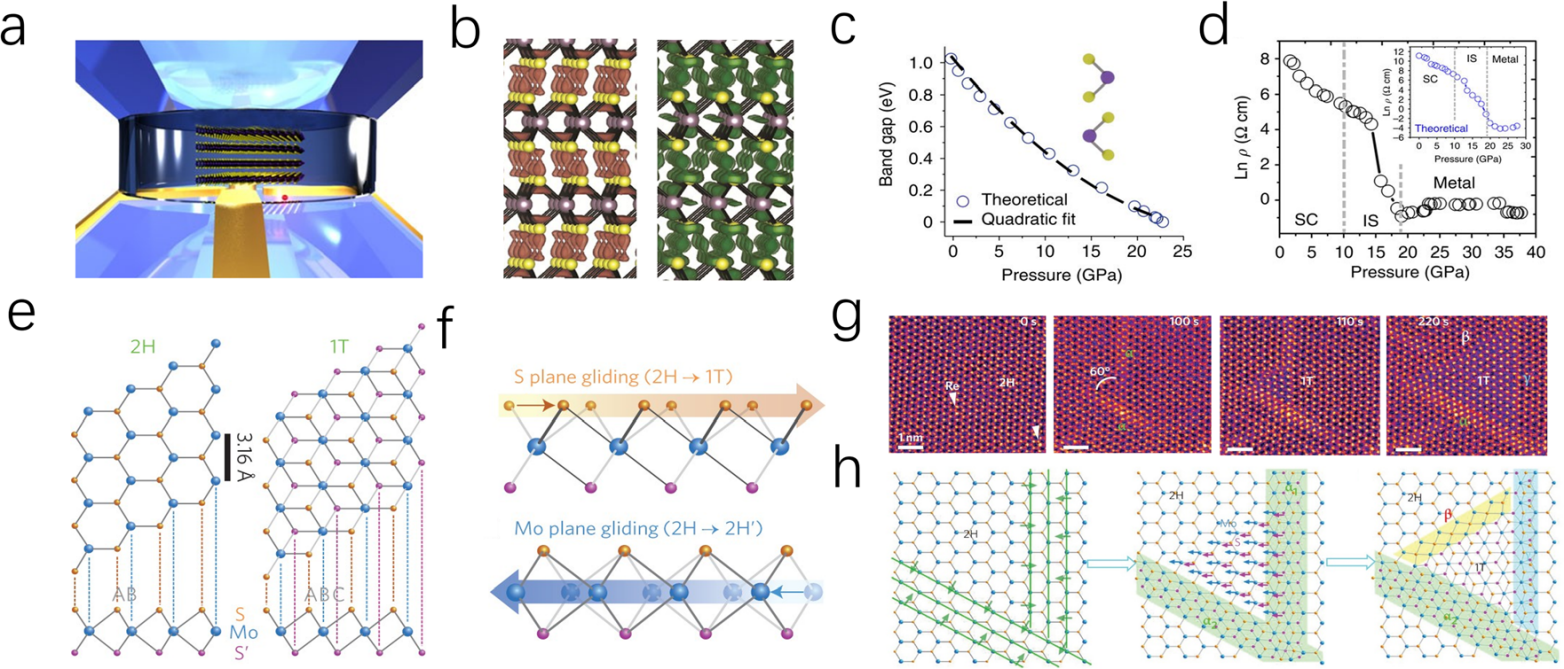
(a) A 3D illustration of multilayered MoS_2_ in a DAC pressure medium for compression experiments. (b) Theoretical charge density isosurfaces of multilayered MoS_2_, charge accumulation (green) and depletion (orange) at the in-plane. (c) Theoretical calculation of the pressure-dependent band gap of multilayered MoS_2_. Inset: the unit cell of MoS_2_. (d) Pressure-dependent electrical resistivity of MoS_2_. Three characteristic regions have been identified: semiconducting (SC), intermediate state (IS) and metallic regions. (Inset) Theoretically calculated pressure-dependent electrical resistivity. (e) Schematic models of single-layered MoS_2_ with 2H and 1T phases in basal plane and cross-section views. Mo, blue; top S, orange; bottom S′, purple. (f) Transformation of 2H to 1T phase caused by S-plane sliding; the sliding of the Mo plane leads to the transformation of the 2H to the 2H’ phase. (g and h) Atomic movements during 2H to 1T phase transformation in single-layered MoS_2_ at *T* = 600 °C.

Beyond controlling the number of layers and stacking patterns, applying local lattice strain or pressure engineering is a powerful approach for fine-tuning the band structure and electrical properties of TMDs. As depicted in Fig. 6a and b, Nayak *et al.* investigated the electronic structure and lattice vibrational dynamics of pristine 2H-MoS_2_ and distorted monolayer 1T-MoS_2_ using diamond anvil cell experiments and DFT calculations.^71,72^ Their study revealed that inevitable lattice distortion occurs, leading to an electronic transition from semiconducting to metallic at a pressure of approximately 19 GPa (Fig. 6c and d). The eventual metallization occurs due to the overlap of the valence and conduction bands, driven by S–S interactions as the interlayer gap decreases. Later, Zhao *et al.* demonstrated the *z*-axis in the MoS_2_ crystal cell is much more compressible than *a*-axis during the compression process, due to the weak vdW interaction between layers.^73^ Therefore, the eventual metallization arises from the overlap of the valence and conduction bands owing to the reduction of interlayer gap, which makes the MoS_2_ improved conductivity and faster HER kinetic process.^74^

In addition to applying crystal pressure, researchers have found that electron beam irradiation can also relieve lattice stress in MoS_2_ by disrupting lattice symmetry. This process causes atoms to migrate to lower energy sites, leading to structural reconstruction. As depicted in Fig. 6e and f, Lin and co-workers employed electron beam irradiation to disrupt the local stress equilibrium within the 2H-MoS_2_ lattice.^75^ This process induced the migration of Mo and S atoms, leading to the formation of a distorted 1T phase at the reconstructed domains. The resulting phase exhibited a triangular morphology with well-defined 2H–1T phase boundaries (Fig. 6g and h). Moreover, Katagiri and colleagues successfully demonstrated phase transformation patterning of MoS_2_ at room temperature using a 160 MeV nm^−2^ electron beam. Due to the interaction between the excited electron beam and the lattice, the 1T/2H in-plane Schottky junctions produced by this technique exhibited a lower barrier (0.13–0.18 eV) than the theoretical value (0.47 eV). Furthermore, the MoS_2_ crystal domains minimized electron scattering and barriers, thereby enhancing electron mobility while improving the kinetics of electrocatalytic reactions.^76^ Fine-tuning the micro-regional configuration and electronic properties of MoS_2_ through intracrystalline stress can promote the formation of unsaturated active sites and induce specific phase structures. However, due to the complexity of the required technology and high equipment costs, this approach is often coupled with theoretical calculations to gain deeper insights into the structural evolution and catalytic activity mechanisms.

## 4 Surface chemical modification

The redistributed electronic landscape of MoS_2_ alters the interactions between the electrocatalytic centers and intermediate hydrogen species when the host catalyst comes into contact with additives (such as atoms, ions, or molecules) or when vacancies are introduced.^77,78^ Surface chemical modification is an effective approach for the regulation of the crystal field and ligand field of MoS_2_, which can greatly affect the physical–chemical properties and significantly improve its catalytic activity. To date, the ligand structure of MoS_2_ can be modulated through vacancy defect, atomic doping, phase transition engineering and heterostructure engineering to achieve significant changes in their electrical nature, magnetic properties, and electrocatalysis performance.^15,40,44,79^ This section provides a detailed discussion of these approaches and highlights several representative examples.

### 4.1 Vacancy defect

**Fig. 7:**
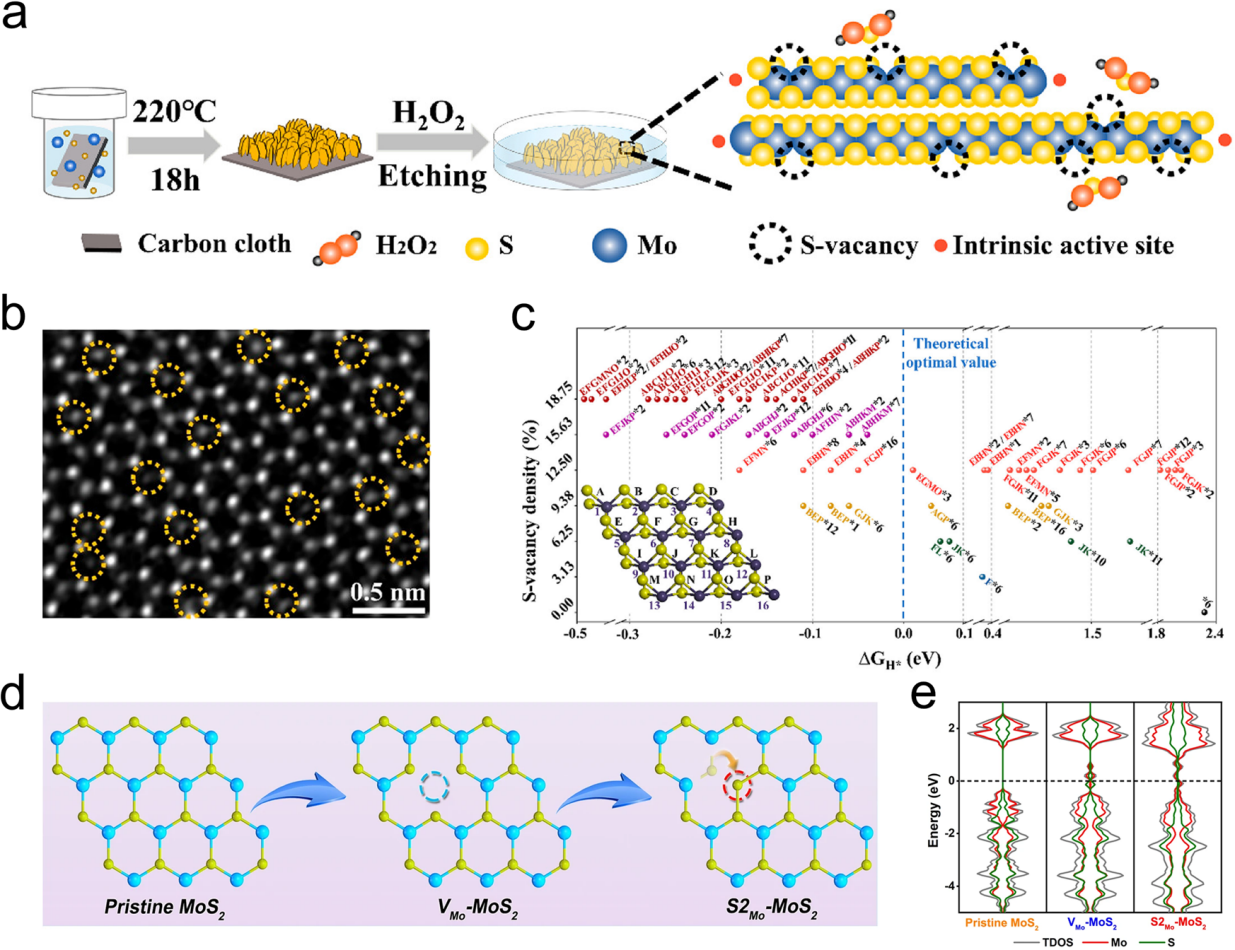
(a) Schematic of the chemical etching process to introduce single S-vacancies. (b) STEM images of CVD-grown single-layer MoS_2_ thin films after etching. The yellow dashed circles represent S vacancies. (c) Free energy *versus* the reaction coordinates of MoS_2_ with diverse S-vacancy states. Inset: corresponding MoS_2_ models in which Mo atoms and the upper S-vacancies are symbolized by the numbers 1–16 and the letters A–P, respectively. (d) Schematic diagram of the formation process of the antisite defect. (e) Total and partial density of states (DOS) of pristine MoS_2_, V_Mo_-MoS_2_, and S2_Mo_-MoS_2_. The Fermi level is set to zero and denoted by the black dotted line.

Introducing vacancy defects is a straightforward and effective strategy for creating additional active sites and fine-tuning the coordination environment of catalytic centers, making it a valuable method for catalyst modification. Typically, these defects are generated by disrupting the crystal periodic structure without the need for external additives.^79^ As depicted in Fig. 7a–c, Wang *et al.* precisely controlled the concentration of S-vacancy on the MoS_2_ surface by H_2_O_2_ etching, revealing the fact that the residual carriers left in S-vacancy prefer transferring into adjacent Mo atoms, which leading the Mo sites stronger H* attraction as a result of de delocalized electrons.^46^ Frenkel-defect (FD) is one of the typical vacancy defects among many related works. For example, Liu Song's group used a simple argon gas annealing treatment to exploit this defect in MoS_2_ and boost hydrogen evolution. It is revealed by DFT calculations that the FD can raise a unique charge landscape and appropriate sites for H* species. By comparison to the single atomic Pt doped MoS_2_ (Pt-MoS_2_), the overpotential of FD-MoS_2_ decreased from 211 mV to 164 mV at a current density of 10 mA cm^−2^.^43^ Subsequently, they prepared antisite defect structures with a concentration of 4% (where two sulfur atoms or a single sulfur atom occupy the position of a molybdenum atom, denoted as S2_Mo_-MoS_2_ or S_Mo_-MoS_2_) within the monolayer MoS_2_ basal plane through calcination in an H_2_/Ar atmosphere, and investigated the role of these antisite defects in promoting the HER process (Fig. 7d). According to DFT analysis, the formation of antisite defects disrupts the original coordination of catalytically S atoms, resulting in a distinctly different and asymmetric charge density distribution in MoS_2_ (Fig. 7e). This change induces a transition from a semiconductor to a metallic state by introducing electronic states at the Fermi level, thereby enhancing H* adsorption.^80^

It is worth noting that specific types of vacancies can be generated by doping with designated heteroatoms, for example, metal dopants are more likely to expose S vacancies. Therefore, fully leveraging the synergistic effects of metal heteroatoms and vacancy defects can lead to remarkable improvements in HER performance. More examples are reflected in alkali metals that have strong interactions with S atoms, such as Zn, Fe, Co, Pd, and Cu.^78,81–83^ The concentration of missing S atoms can be easily controlled by controlling the amount of alkali metals.^82,84^ In addition, as low-loaded noble metals are expected to boost HER performance, the synergistic effect of noble metals and vacancy defects is highlighted in reducing the H* adsorption/desorption energy barrier and improving HER performance.

### 4.2 In-plane substitution

In-plane substitutional doping of MoS_2_ can be achieved through two strategies: replacing host Mo and S atoms, which is the main way to achieve in-plane charge rearrangement.^85^ Non-metal elements such as C, N, O, and P. are used as the main in-plane substituents due to their easy operation and low cost.^86–89^ Ge and co-authors replaced the S atoms in MoS_2_ with O atoms through a simple wet chemical method. The high electronegativity of O induced electron outflow from the adjacent S atoms, thereby constructing an efficient HER 1T-MoS_2_ catalyst. Additionally, O atoms tend to form hydrogen bonds with H_2_O in the electrolyte, enhancing the adsorption and dissociation of H_2_O on the catalyst surface. While the high electronegativity of non-metal elements is an effective strategy for driving charge transfer from host S atoms, an excessive electronegativity gap can lead to localized charge accumulation and slow H* desorption process.^88^ Jiang *et al.* addressed this issue by Co-doping MoS_2_ with both Se and O atoms, whose electronegativities are 2.55 and 3.5, respectively. Studies have shown that Se–O co-doped MoS_2_ exhibits moderate energy level splitting due to the synergistic effect of Se–O on the electronic structure. Furthermore, the smaller atomic diameter of Se can induce lattice distortion by replacing partial S atoms, resulting in Se-MoS_2_ electrocatalyst exhibiting a low Tafel slope of ∼47 mV dec^−1^ under acidic conditions.^90^

**Fig. 8:**
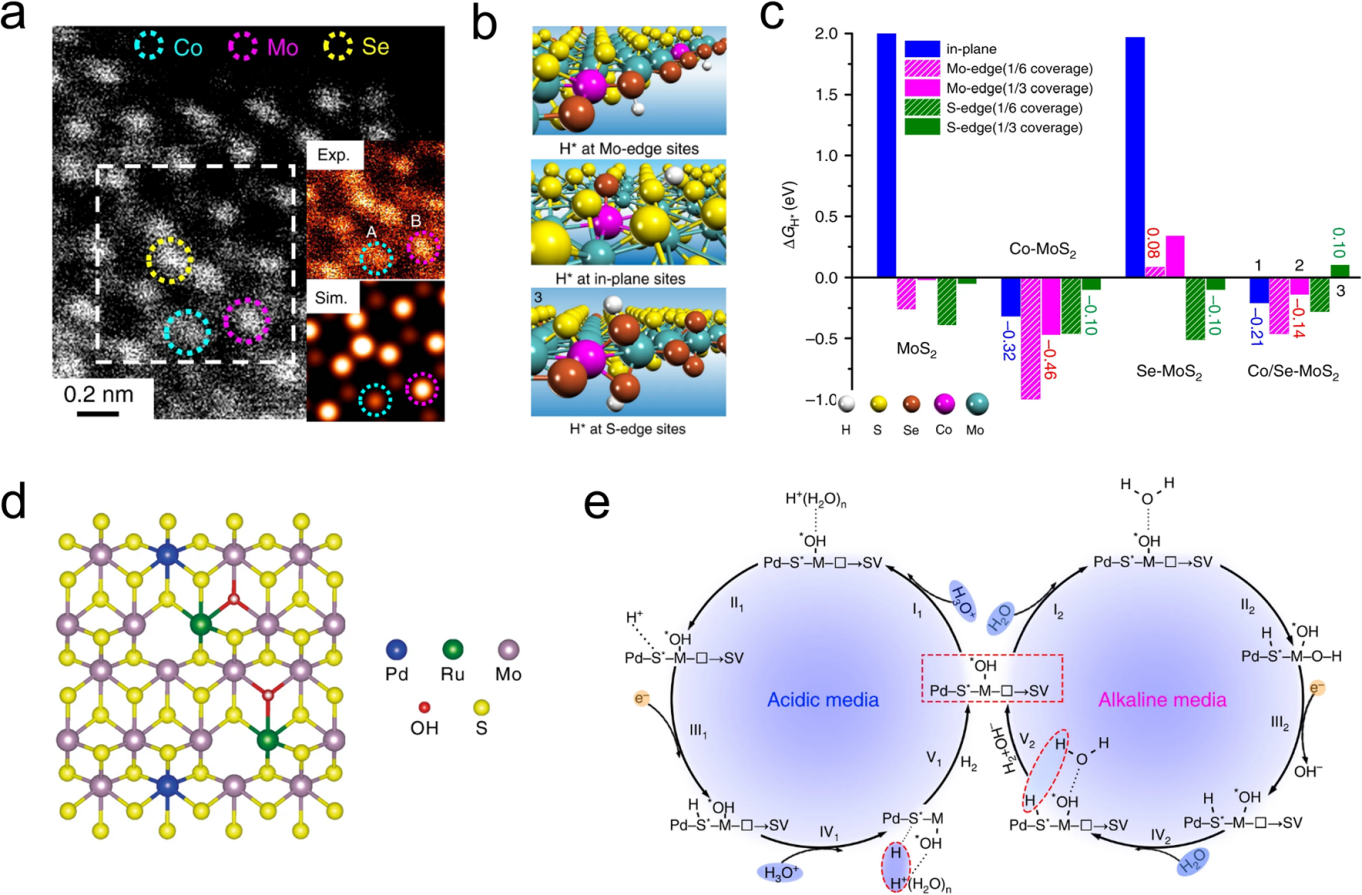
(a) Atomic resolution HAADF-STEM image of Co/Se-MoS_2_-NF. Inset: magnification of the white dashed area in (a). (b) H* adsorption structures at the (b) Mo edge, (c) plane, and (d) S edge positions of Co/Se-MoS_2_. (c) H* adsorption free energies (Δ*G*_H*_) at the plane, Mo edge, and S edge positions of pure MoS_2_, Co-doped, Se-doped, and Co/Se co-doped MoS_2_, denoted as MoS_2_, Co-MoS_2_, Se-MoS_2_, and Co/Se-MoS_2_, respectively. The hydrogen coverage at the Mo edge is defined based on the terminated S or Se monomer positions, while the hydrogen coverage at the S edge is defined based on the terminated S or Se dimer positions, which are the active sites for HER. (d) Schematic diagram of Pd and Ru dual-doped MoS_2−*x*_OH_*y*_ phase. The blue, yellow, purple, green, and red spheres represent Pd, S, Mo, Ru, and O atoms, respectively. (e) (Left) HER mechanism of Pd, Ru-MoS_2−*x*_OH_*y*_ in acidic media; (Right) HER mechanism of Pd, Ru-MoS_2−*x*_OH_*y*_ in alkaline media.

To avoid the violent host–guest electron interaction, it is feasible to further awaken the inertness of metal sites. The synergistic effect of metal-nonmetal diatomic is developed to stimulate the utilization of MoS_2_. Transition metals (TMs), with their abundant d-orbital electron states and spin magnetism, are well-suited for adjusting the Fermi level of MoS_2_ by forming s–p hybridized orbitals with S atoms.^40^ As depicted in Fig. 8a, Deng and coworkers synthesized a Co and Se co-doped MoS_2_ nanostructured foam electrocatalyst (Co/Se-MoS_2_-NF) by confining Co atoms within the sublayer and Se atoms within the surface lattice of MoS_2_ using a one-step hydrothermal method. In this configuration, Co atoms serve as substitutional dopants for sublayer Mo, activating the inert S sites, while Se atoms are confined in the surface to stabilize the basal plane. The synergistic effect of Co and Se is evident in the optimized thermodynamics and structural stability of the HER sites (Fig. 8b and c). The near-zero free energy significantly accelerates the formation of H_2_ formation, while the balanced electronic structure ensures the long-term stability of the active sites. Tests revealed that Co/Se-MoS_2_-NF exhibited a low Tafel slope of 67 mV dec^−1^ and a long-term performance over 360 h at a high current density of 1000 mA cm^−2^ in 0.5 M H_2_SO_4_.^91^

More recently, bimetallic atoms pair has also been widely used for the tandem regulation of the HER process. Li *et al.* developed a Ni, Co bimetallic atoms dual modified MoS_2_ catalyst (Ni, Co-MoS_2_) to enhance HER activity in alkaline solutions. The study showed that Ni atoms serve as initial activation sites for the H_2_O, while Co atoms facilitate the adsorption and desorption kinetics of H intermediates by influencing the electronic structure of the neighboring S. The Ni, Co-MoS_2_ catalyst exhibited a lower Tafel slope of 78 mV dec^−1^.^92^ Similarly, Han and coauthors developed a Cu and Pd dual-doped MoS_2_ catalyst (Cu–Pd-MoS_2_) using a two-step approach. The study showed that Cu enhanced the conductivity, while Pd facilitated the phase transition from 2H to 1T phase, thereby improving both the electron transfer and the number of active sites.^93^ Additionally, Luo *et al.* engineered a di-anionic surface (Pd, Ru-MoS_2−*x*_OH_*y*_) on MoS_2_ to modulate the HER mechanism (Fig. 8d). The study demonstrates that by replacing S sites with –OH anions, a strong non-covalent hydrogen interaction was established, effectively attracting hydronium ions and water molecules. Furthermore, the hydroxyl network works synergistically with adjacent metal atoms (M–OH), overcoming the kinetic barriers associated with water dissociation in alkaline media (Fig. 8e). As a result, the Pd, Ru-MoS_2−*x*_OH_*y*_ catalyst exhibits superior kinetic performance in both acidic and alkaline conditions, with Tafel slopes of 85 mV dec^−1^ and 121 mV dec^−1^, respectively.^94^

### 4.3 Embedding doping

**Fig. 9:**
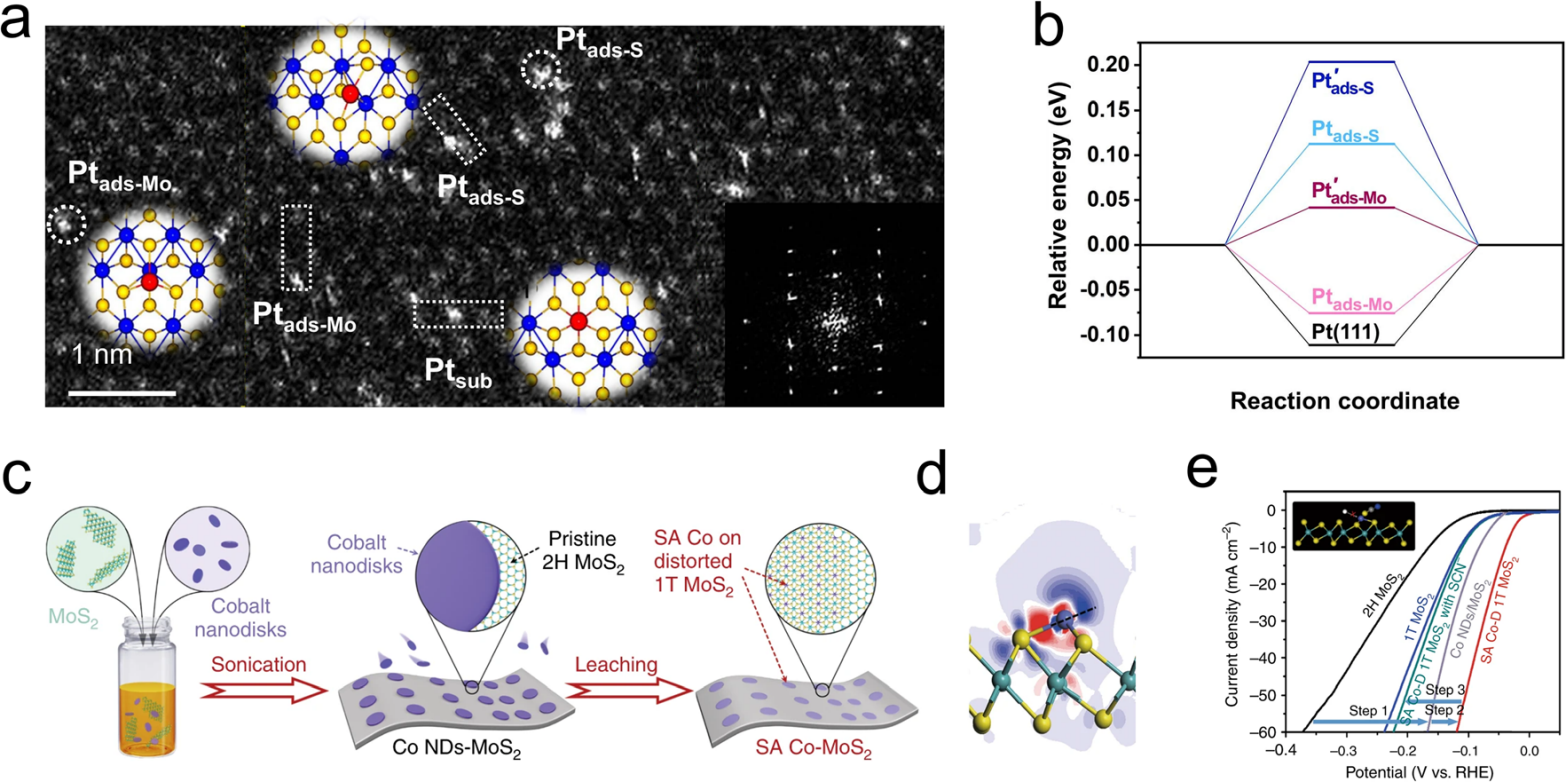
(a) Atomic-resolution HAADF-STEM image of s-Pt/1T′-MoS_2_ showing the isolated Pt atomically dispersed on 1T′-MoS_2_ NS. Three kinds of single-atomically dispersed Pt, denoted as Pt_sub_, Pt_ads-S_ and Pt_ads-Mo_, respectively, are enclosed by the white dotted rectangles. (Inset) Simulated atomic structure diagram and corresponding FFT pattern. (b) Calculated Δ*G*_H*_ diagrams for HER. (c) Schematic diagram of the fabrication process for SA Co-D 1T MoS_2_. (d) The electron density difference of 3 × 3 case. (e) HER polarization plots of MoS_2_, 1T MoS_2_ prepared by lithiation, Co NDs/MoS_2_ and SA Co-D 1T MoS_2_ without and with SCN-ions. The inset shows that the cobalt HER active centers are blocked by SCN-ions.

Driven by the high chemical potential of the reactants, the interstitial spaces within the MoS_2_ lattice become occupied by free atoms.^95–97^ These structural additives can subsequently modify the catalytic behavior by tuning the Fermi level and the density of states of neighboring atoms. Interestingly, these additives may directly alter the reaction pathway or indirectly influence the chemical process by facilitating the proper adsorption of H* by adjacent atoms.^98,99^ However, the instability of these structural additives and their strong dependence on the coordination environment pose challenges to heterogeneous catalysis. Noble metal single atoms are widely considered to be excellent regulators of HER, and the potential candidates for heterotopic doping as well. As depicted in Fig. 9a, Shi and coauthors utilized a photoreduction method to load dispersed Pt single atoms onto a 1T′-MoS_2_ catalyst. Structural characterization revealed that the loaded Pt atoms predominantly exist in three forms: substitutional Pt (Pt_sub_), Pt adsorbed on top of S (Pt_ads-S_), and Pt adsorbed on top of Mo (Pt_ads-Mo_). Among these, the Pt_ads-Mo_ sites exhibited nearly zero free energy for hydrogen evolution (Fig. 9b), resulting in a mass activity of 85 ± 23 A mg_Pt_^−1^ for the s-Pt/1T′-MoS_2_ catalyst at an overpotential of −50 mV.^100^

Utilizing the d-orbital effects of transition metal Co, Qi *et al.* employed cyclic voltammetry (CV) to deposit Co nanodisks (NDs) onto the MoS_2_ surface (Fig. 9c). The incorporated Co atoms were found to occupy positions atop Mo atoms rather than at MoS_2_ defects or edge sites, leading to the formation of a distorted 1T phase (Fig. 9d). Research indicates that these single Co atoms act as active centers, directly participating in the binding of H intermediates on the MoS_2_. The resulting Co-D 1T MoS_2_ demonstrated outstanding HER performance as illustrated in Fig. 9e, with a low Tafel slope of 32 mV dec^−1^ and long-term stability over 2400 h.^101^ To develop a highly stable interstitial doping system, Zhou *et al.* incorporated carbon atoms with an ultra-small atomic radius and high chemical potential into the sublayer interstices of MoS_2_. The study focused on the structure–performance relationship of the HER in the interstitial carbon-doped MoS_2_ electrocatalyst (C_ia_-MoS_2_). Through hydrothermal synthesis, the atomic C was strategically embedded into the fcc sites of the MoS_2_ cell, resulting in a favorable local charge landscape and a 1T phase that promoted HER activity. Unlike previous studies, the C_ia_ did not directly accelerate the HER process but instead served as a structural stabilizer for the emerging metallic phase. The synthesized C_ia_-MoS_2_ catalyst showed an 81% reduction in overpotential at 10 mA cm^−2^ compared to pristine MoS_2_.^99^ The introduction of heterotopic structural additives is increasingly being developed to enhance the stability of active phases, or provide additional adsorption sites and conductivity. These atypical doping sites can effectively alter reaction pathways and are generally suitable for additives with high dispersion, small size, and large chemical potential differences from the host catalyst.

### 4.4 Heterostructure construction

**Fig. 10:**
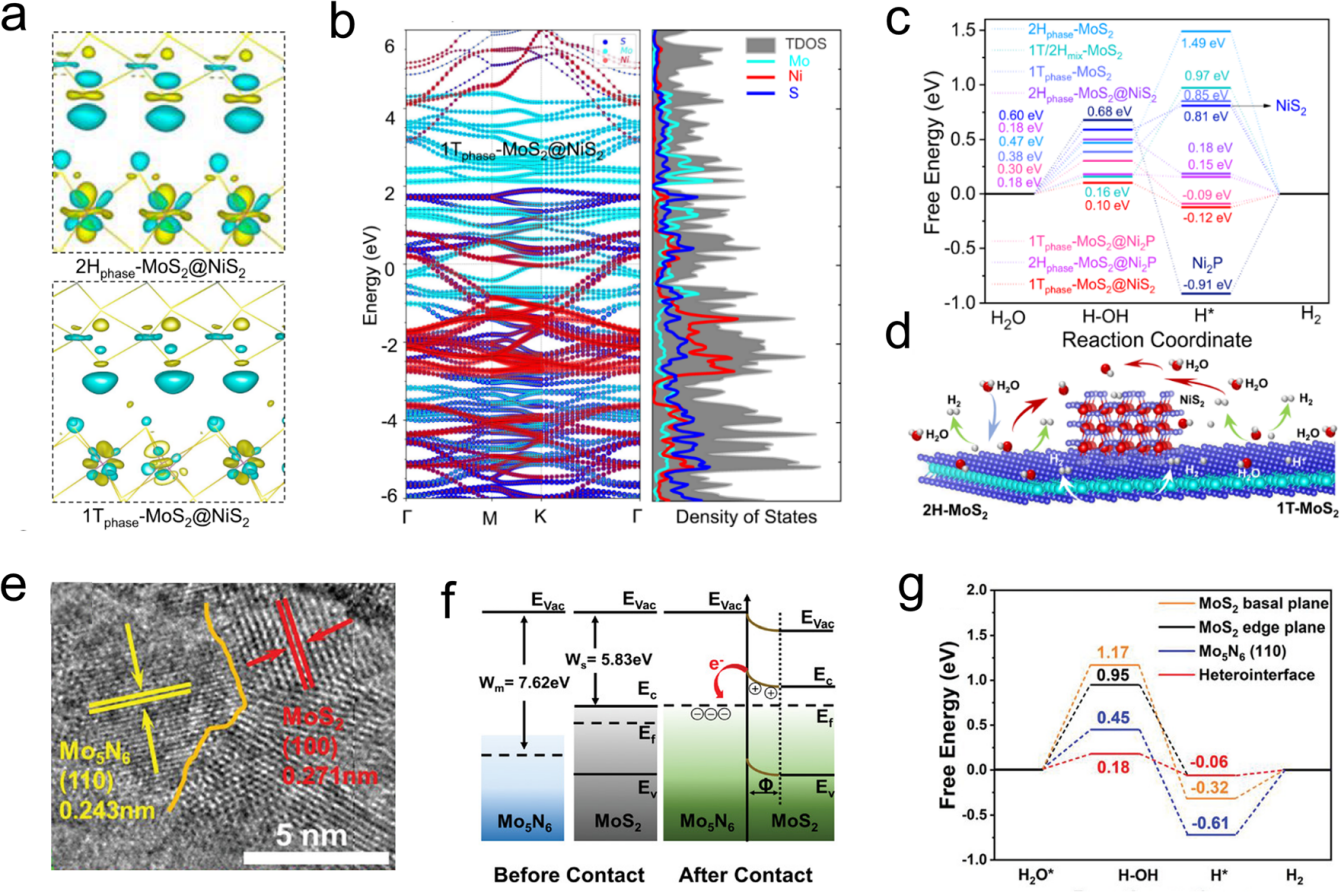
(a) The deformation of the electronic density of 2H_phase_-MoS_2_@NiS_2_ and 1T_phase_-MoS_2_@NiS_2_ interface, in which yellow/green isosurfaces correspond to positive/negative spin densities (0.00295308 e Å^−3^). (b) Band structure and density of states (DOS) for 1T_phase_-MoS_2_@NiS_2_. (c) Free-energy diagrams for HER on the 2H_phase_-MoS_2_, 1T_phase_-MoS_2_, pure Ni_2_P, pure NiS_2_, 1T/2H_mix_-MoS_2_, 2H_phase_-MoS_2_@Ni_2_P, 2H_phase_-MoS_2_@NiS_2_, 1T_phase_-MoS_2_@NiS_2_ and 1T_phase_-MoS_2_@Ni_2_P interface edge. (d) Schematics showing water activation, *H intermediate formation and hydrogen generation on multi-heterojunction electrocatalysts. (e) The HR-TEM images of the Mo_5_N_6_-MoS_2_ heterojunction by in-plane chemical bonding of MoS_2_ (100) basal planes and Mo_5_N_6_ (110) plane. (f) Schematic diagrams of the energy band of Schottky-type Mo_5_N_6_-MoS_2_ heterojunction (*E*_vac_ = vacuum energy, *E*_c_ = conduction band, *E*_v_ = valence band, *E*_f_ = Fermi level, *E*_g_ = energy gap, *W* = work function, and *Φ* = depletion region). (g) Energy profiles for water dissociation and free energy diagrams for HER on 2H-MoS_2_ basal plane, 2H-MoS_2_ edge plane, Mo_5_N_6_ (110), and Mo_5_N_6_-MoS_2_ heterointerface.

When 2D materials are combined through regions with different atomic arrangement orientations, rich physical and chemical properties have emerged, and the transition zone is called domain wall.^102,103^ Fansy phenomenon can be seen near the dislocation region, *e.g.*, effective charge transfer, impurity band, intermediate structure and inhomogeneous electronic structure.^104,105^ Hong *et al.* developed a chemical vapor deposition technique to synthesize a single-layer MoS_2_ with a 1T–2H heterostructure, in which the 1T phase was as high as 61.5%. DFT calculations revealed that the strong electronic coupling was observed at the heterogeneous interface, and the electrons spontaneously transfer from the 2H phase to the 1T phase. This phenomenon was lead an increased electronic density around 1T phase while a depletion of electrons around the 2H phase, causes the transition region a low HER barrier (−0.156 eV) and fast kinetic process (Tafel slope is 78 mV dec^−1^).^106^ Along this line, more heterogeneous structure can be organized by MoS_2_ and other materials. Such as 1T_0.81_-MoS_2_@Ni_2_P HER catalyst studied by Liu *et al.* Firstly, the high electronegativity of P drives pristine MoS_2_ partial shift to 1T phase; then the electrons were transfer from the MoS_2_ into Ni_2_S or Ni_2_P regions, leading a rich electronic density environment around the domain (Fig. 10a and b). According to the rich heterogeneous interfaces, the as synthesized 1T_0.81_-MoS_2_@Ni_2_P catalyst accelerates the H_2_O activation, as well as the generation of intermediate H species (Fig. 10c and d), which possesses an extremely low overpotential in acidic (38.9 mV) and alkaline (95 mV) solutions at 10 mA cm^−2^, corresponding to a considerable Tafel slope of 41 and 42 mV dec^−1^, respectively.^107^

Constructing in-plane contact between metal and semiconductor is another effective approach to generate a built-in electric field using Schottky barriers. Pi *et al.* prepared the Mo_5_N_6_-MoS_2_ heterojunction nanosheets (Mo_5_N_6_-MoS_2_/HCNRs) on conductive hollow carbon nanoribbons by a simple hydrothermal method (Fig. 10e). The results showed that there was a Fermi level difference in the metal/semi-contact, which led to the electron transfer from 2H-MoS_2_ to Mo_5_N_6_ (Fig. 10f). In addition, the lattice dislocations led to abundant defective sites, which increased the number of active sites and charge transfer process, giving the catalyst a low overpotential of 53 mV at 10 mA cm^−2^ and a Tafel slope of 37.9 mV dec^−1^ (Fig. 10g).^108^ Heterogeneous structures are typically characterized by abundant crystal domains, and fully utilizing these interfaces can significantly enhance the electrical properties of catalysts. In addition, domain walls are often available for H* species adsorption and activation, which play a role in increasing active sites.

## 5 Interlayer coupling engineering

Layered intercalation is an effective method to modulate interlayer coupling and crystal field in MoS_2_.^109^ By widening the vdW gap, the crystal field of MoS_2_ switches from tetrahedral to octahedral coordination, leading to a re-splitting of energy levels.^23,77^ In addition, direct growth of MoS_2_ on metal support has been emerging as a novel approach to synthesize catalytic complexes. Taking advantage of the Fermi level difference between metals and semi, a large number of electrons can be transferred to MoS_2_ through metal supports, thus promoting the reconstruction of active centers.^22,110–112^ This section focuses on two typical interlayer couplings and their applications for enhancing the HER performance of MoS_2_ catalysts.

### 5.1 Modulation of vdW gap

**Fig. 11:**
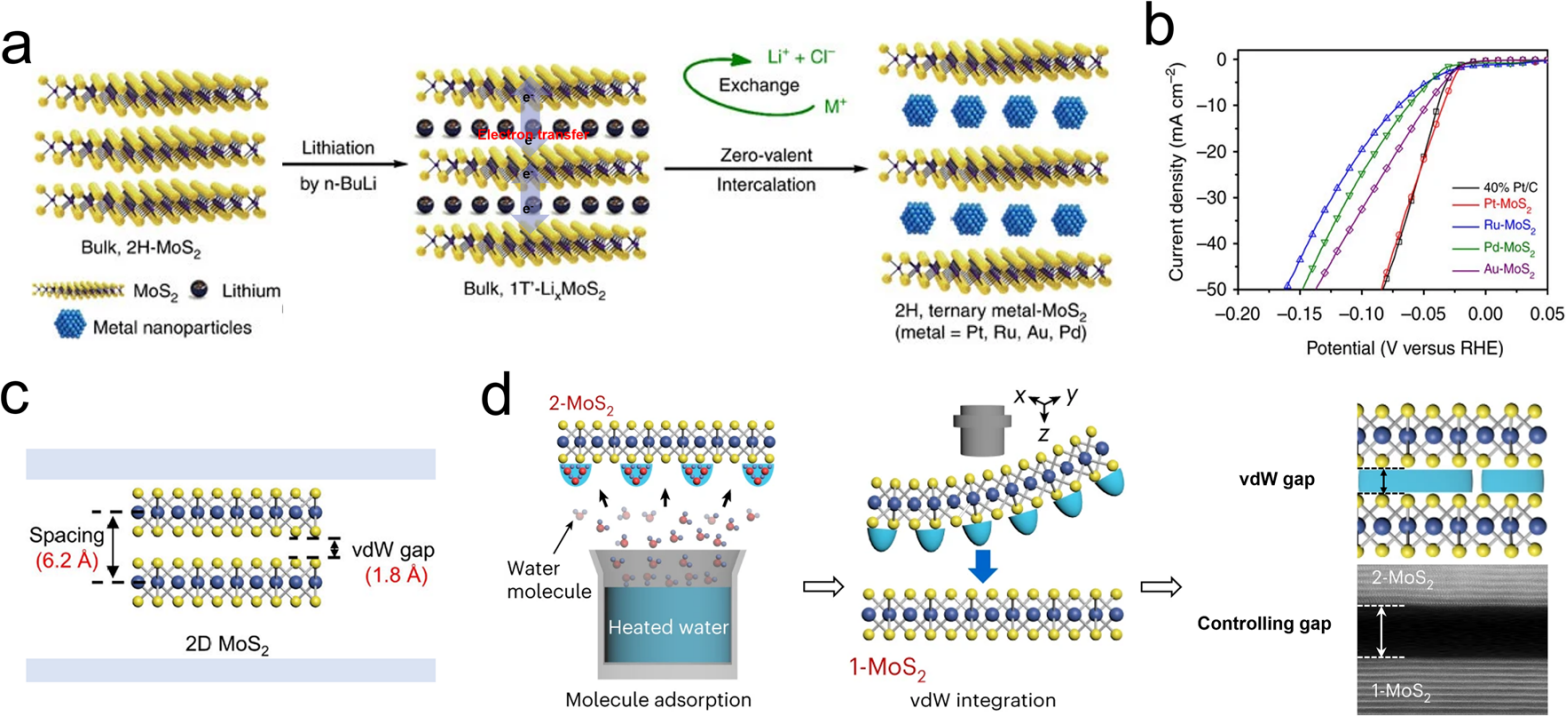
(a) Zero-valent intercalation of metal nanoparticles by an *in situ* reduction strategy. (b) LSV curves of metal intercalated MoS_2_ catalysts. (c) Intrinsic van der Waals distance and van der Waals gap of MoS_2_ (van der Waals distance = 6.2 Å, van der Waals gap = 1.8 Å). (d) Schematic illustrations of the vdW gap engineering achieved by adsorption of water molecules, and the cross-sectional STEM image of the vdW interface formed by vdW gap engineering, showing a clear vdW gap while maintaining atomically sharp and clean interfaces. Scale bars, 5 nm (STEM image).

2D layered MoS_2_ are attracting each layer by weak physical vdW interaction (shown as Fig. 11c), and the properties of MoS_2_ catalysts directly endued by the height of vdW gap.^113^ Existing strategies for expanding the interlayer gap are mainly based on electrochemical intercalation techniques, whereby additive ions, molecules, and nanoparticles are driven into the vdW gaps of MoS_2_.^34,114^

Alkali metal ions are popular in modifying the vdW gap and chemical properties of MoS_2_, as they have extremely small diameter and low ionization potential.^115^ For example, Cui *et al.* reported a vertically aligned MoS_2_ electrocatalyst intercalated by Li^+^ for rapid HER. First of all, the Li^+^ acts as a gap extender, which directly changes the vdW interaction and alters the electronic properties. Secondly, Li^+^ transfers extra electrons to MoS_2_ due to its high reducibility, thus changing the spin–orbit coupling of Mo 3d orbital. Last, it was proved that the 2H phase MoS_2_ shifts into 1T phase when the concentration of Li^+^ is over 0.28.^19^ In addition, the capillary action of MoS_2_ powder and the molten K^+^ was used for the modulation of the vdW gap. It was reported the added K atoms can be driven into the vdW gaps to prepare complex artificial superlattices, due to the difference between the electron affinity of MoS_2_ (4.45 eV) and the ionization potential of K^+^ (4.34 eV).^26^ Based on this, as depicted in Fig. 11a and b, Chen *et al.* achieved the intercalation of several metal nanoparticles into a Li^+^-intercalated 1T′ Li_*x*_MoS_2_ catalyst by *in situ* reduction of noble metal ion precursors. In this process, the wider vdW gaps provided channels for the diffusion of target noble species, endowing the novel catalyst with a higher redox potential. Unlike the in-plane doping, the intercalation of alkali metal ions species reacts with surface S atoms and forms M–S bonds (M = K, Na, Pt, Au), which suppresses electron scattering and maintains a high electron concentration around the d orbitals of Mo.

However, the introduction of alkali metal ions was realized through a hysteresis diffusion-limited process, leading to a fuzzy crystalline phase and reduced structural stability.^116^ To overcome these drawbacks, a self-intercalation approach was developed by Loh and his colleagues, where the host metal atoms move into the vdW gaps by exploiting their high chemical potential. It was found that the self-driven intercalant introduces additional spin-split bands, which can modulate the electronic properties of the carrier transport.^117^ An advanced solution was proposed by Zou *et al.*, who exploited the vdW gaps to confine small pre-adsorbed water molecules. By controlling the saturation vapour pressure of water, the ‘vdW water gap’ was monotonic increasing up to 53.6 Å (Fig. 11d). This gap-engineering approach highlights the potential of vdW water gaps to modulate electronic properties and more utility for diode and electrochemical devices.^113^

### 5.2 Semiconductor/metal contacting

**Fig. 12:**
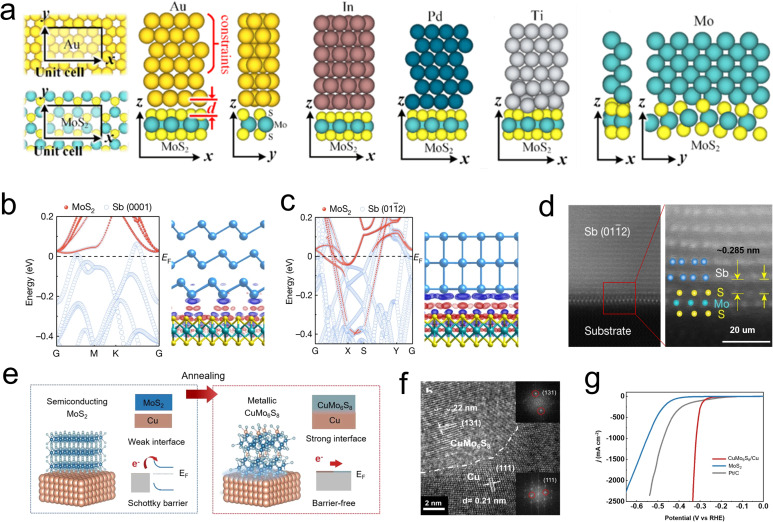
(a) Optimized geometries of top contacts to MoS_2_: Au-MoS_2_ (in different views), In-MoS_2_, Pd-MoS_2_, Ti-MoS_2_, Mo-MoS_2_ (in different views). d is defined as the physical separation (the *z* component of the nearest core-to-core distance between the metal atoms and the chalcogenide atoms). (b) Atomic projected electronic band structure of Sb (0001)–MoS_2_ contact and charge density near junction EF. (c) Atomic projected electronic band structure of Sb (0112)–MoS_2_ contact and charge density near junction EF (red, positive; blue, negative). (d) (Left) Cross-section HAADF-STEM image of the Sb (0112)–MoS_2_ contact. Scale bar, 2 nm. (Right) Zoom-in atomic-resolution image from the red box in (c). The Mo, S and Sb atoms are overlayed on the image. The vdW gap of 0.285 nm is marked. Scale bar, 1 nm. (e) A schematic showing the preparation process of CuMo_6_S_8_/Cu electrode. (f) Cross-sectional high-resolution transmission electron microscopy (HRTEM) zoom-in view of the CuMo_6_S_8_/Cu electrode. The inset of h is the corresponding fast Fourier transform patterns of CuMo_6_S_8_ (top) and Cu (bottom). (g) Polarization curves of three electrodes including CuMo_6_S_8_/Cu, MoS_2_, and Pt/C. All tests are done in 1 M KOH at a scan rate of 1 mV s^−1^ with 85% *iR* correction.

Generally, 2D films are grown on support like metal (*e.g.*, Au, Pd, Cu, and Mo) or oxides (*e.g.*, mica, sapphire, and silica) for robust action.^20^ Much effort has been put into exploring various supports, and a strong interaction between Au foil and MoS_2_ films has been discovered in 2014. It is revealed that the electronic coupling effects between Au and MoS_2_ and eliminate the charge transfer resistance, thus promote the catalysts reaching a high exchange current density of 38.1 μA cm^−2^.^59^ Obviously, semi/metal contacting effects play a vital role in design Schottky barrier and regulate the conductivity. Voiry *et al.* pioneered to concern the relationship of semi/metal contact effects on HER activity,^22^ and Charlie Tsai and coauthors studied how different supports influence the Gibbs free energy of Mo edge sites. Their results show that the HER reactivity of edged-Mo sites can be influenced by tuning the contact effects between semi-MoS_2_ films and different metal supports, where the electronic interaction induced Fermi level shift and reduced interface resistance.^118^ Along this line, as depicted in Fig. 12a, several metals such as In, Ti, Au, Pd, and Mo were used as simulated support to contact with the surface and edges part of monolayer MoS_2_. The orbital overlap, Schottky barrier, and tunnel barriers of TMDs and metal contact effects were disclosed by DFT calculations. Interestingly, the separate distance (d) of the metal-edge is much smaller than the distance of metal-surface in all combinations, thus strong covalent bonds can form between the metal and edge site. In addition, an atomic orbital overlap of sulfides and metal can be found in each case, which may lead to a reduction of the Schottky barrier and inner resistance of the monomer catalyst.^119^

More recently, Li *et al.* studied the contact effect between Sb with different plane orientations Sb (0001) and Sb (0112) prepared by electron beam evaporation and monolayer MoS_2_ (Fig. 12b–d). It is observed that the Mo d orbital has a large real-space overlap with the Sb (0112) p_*z*_ orbital in the vertical direction, resulting in strong Sb (0112)–MoS_2_ hybridization and forcing the interface resistance to approach the quantum limit. In contrast, no obvious electron localization was observed between Mo d orbital and Sb (0001) p orbital.^120^ This work emphasizes the significance of crystal plane orientation in the preparation of zero contact resistance composite catalyst. Directly construct carrier pathway between metal support and MoS_2_ active component also considered an effective method to tune semi/metal contact interface. For example, Shin and Liu developed a highly mechanically stable novel electrocatalyst by growing MoS_2_ nanosheets on a self-support Cu foil, where the MoS_2_ are vertically embedded in the Cu (Fig. 12e). Differently, there is a composite region between the MoS_2_ and Cu, Cu_*x*_Mo_6_S_8_ (CMS), which is known as the Chevrel phase (Fig. 12f). It was shown that the CMS region serves as an effective charge transfer channel to promote the carriers transfer into the MoS_2_ active component, thus exhibiting a low overpotential of ∼334 mV and sustained stability of over 100 h at a current density of 2500 mA cm^−2^ (Fig. 12g).^121,122^ Similar studies were reported by Liu's group, who achieved *in situ* growth of a vertically oriented TaS_2_ active layer on a metallic Ta by an oriented solid-phase synthesis method. It was confirmed that electrons can directly inject into TaS_2_ layers without passing the vdW gap. Additionally, this novel integrated structure demonstrates excellent mechanical stability, with the Ta/TaS_2_ catalyst operating for over 200 hours while maintaining a low overpotential of 398 mV at a high current density of 2000 mA cm^−2^. These advances pave the way to apply this monomer catalyst to boost HER process.^123^

## 6 Challenges and outlooking

**Fig. 13:**
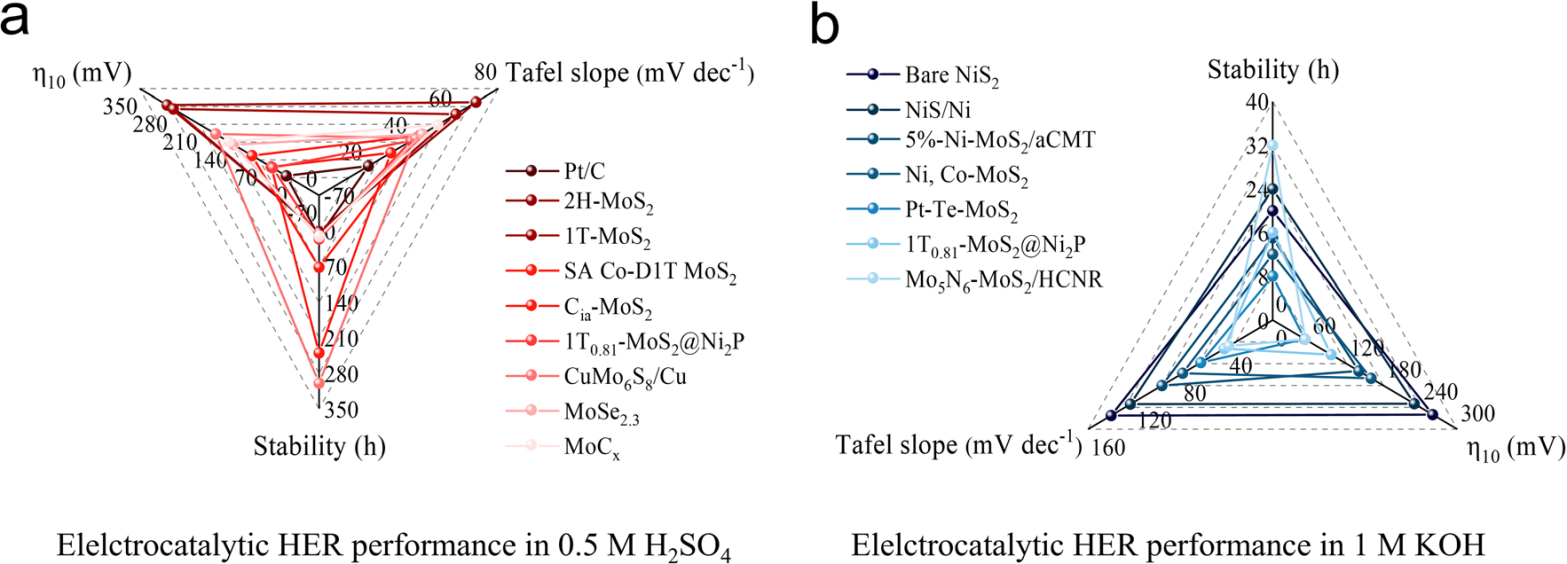
HER performance of various catalysts under (a) acidic conditions and (b) alkaline conditions.

**Fig. 14:**
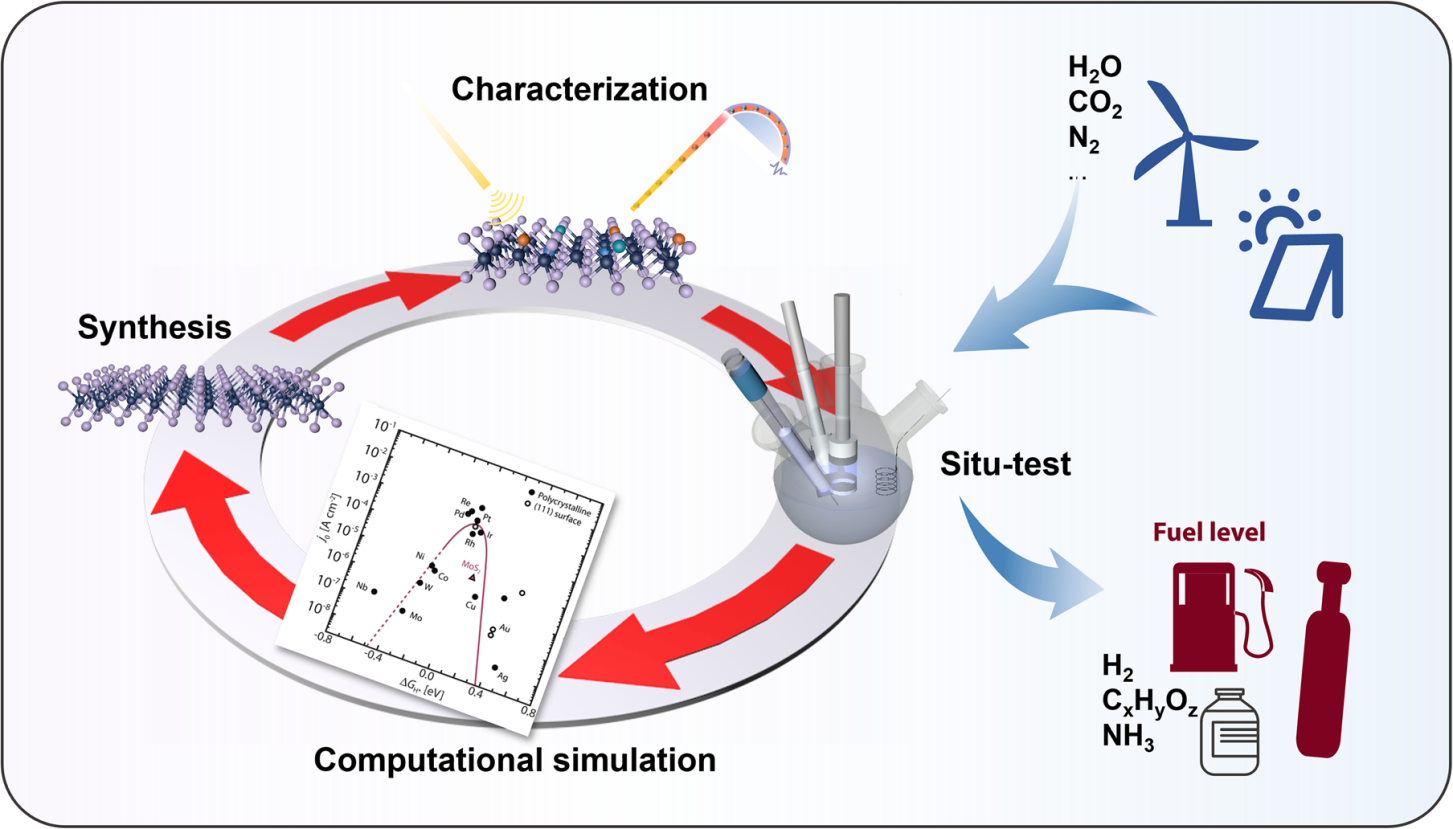
The potential development researches from modern theoretical analysis, advanced synthesis and characterization, and possible applications in electrocatalysis for MoS_2_.

Although the unsaturated electronic structure is regarded as the key feature of MoS_2_ for achieving efficient HER, the long-term electrocatalytic stability is often compromised by the emergence of active structural configurations. Fig. 13a and b illustrate some common cases associated with HER performance. Based on current electrocatalytic test data, it is evident that TMDs based on Mo element tend to perform better under acidic conditions,^99,101,107,122,124–128^ whereas catalysts containing Ni element exhibit greater durability in alkaline environments.^92,107,108,129–132^ These findings provide valuable guidance for selecting appropriate catalysts for large-scale HER processes. As the study of constructing and regulating the structure–activity relationship of MoS_2_-like catalysts at the atomic level is still in its early stages, we propose several research topics to assist researchers to navigate through the scientific opportunities in this area (Fig. 14).

### 6.1 Electronic structure evolution of central metal during electrocatalysis and decay

The evolution of the electron states of d-orbital of the central metal site in transition metal sulfide catalysts during electrocatalysis remains a significant challenge.^133^ Some studies have reported that utilize low-temperature or *in situ* characterization approaches can allow for the semi-quantitative analysis of the local electronic structure and unsaturated states of transition metals. In a reaction process involving electrons transfer, electron transitions within the central metal, electron donation from dopants to the central metal, and the transfer of delocalized electrons between the central metal and ligands can alter the redox properties of the metal sites, thus, the formation, adsorption, or desorption of intermediates and products. Unlike other catalytic mechanisms, the presence of additional energy from magnetocaloric effects associated with intermediate or high-spin electrons could enhance reaction kinetics or intermediate mobility. This could lead to the appearance of “mirages” in electrocatalytic reactions that thermodynamic theory would deem impossible. Hence, researches on the influence of spin effects in transition metal catalysts on reaction activity and selectivity may become a critical focus in future catalysis research.

### 6.2 Role of unsaturated electronic structure in the interaction between reactants/products and catalyst centers

Unsaturated electronic structures are typically accompanied by unpaired electrons, which significantly impact the magnetic properties and polarity of local catalytic centers. As a result, investigating the role of electron deficiency in spin states, magnetism, polarity, and conductivity in MoS_2_ could offer valuable insights into reaction mechanisms and Gibbs free energy pathways, especially in the context of MoS_2_ catalysts applied to more complex reactions. For instance, when products are present in a non-polar bubble state, adjusting the polarity of catalytic centers may be crucial for enhancing the desorption and diffusion of bubble-phase products. Despite bubble trouble becomes a growing focus on this topic in large-scale HER processes, research from this perspective remains limited.

### 6.3 Advantages of metal/semiconductor contacts in constructing highly active electronic structures

In heterogeneous electrocatalysis, harnessing the interfacial contact effects between supported metals and semiconductor catalysts to modulate band positions, charge transfer, and structural stability of electrocatalysts remain a significant challenge. Recent studies suggest that as the van der Waals gap between 2D layered catalysts narrows, it can promote the formation of new chemical bonds or delocalized electrons, thereby creating internal pathways for electron transfer. Thus, exploring and harnessing the interfacial contact effects between supported metals and semiconductor catalysts may offers a promising “two birds with one stone” strategy for monomer catalysts in electrocatalysis-simultaneously improving catalyst stability and optimizing the active electronic structure.

### 6.4 Advanced characterization methods to study unsaturated electronic structures

In the exploration of unsaturated electronic structures in 2D materials (such as MoS_2_), advanced characterization methods are crucial. Commonly used techniques include Raman spectroscopy, X-ray absorption spectroscopy (XAS), X-ray photoelectron spectroscopy (XPS), and electron paramagnetic resonance (EPR). These approaches provide valuable information about electronic structure, chemical environment, and unpaired electrons, but they face challenges when applied to light-sensitive 2D materials. For instance, while Raman spectroscopy is sensitive to unsaturated electronic structures, the signals are often weak at low-energy excitation source and susceptible to interference from fluorescence. XAS and XPS can offer semi-quantitative data on electronic states, but they mainly capture average structures, making it difficult to precisely characterize local defects or active sites. Future advancements may involve combining high-angle annular dark field scanning transmission electron microscopy (HAADF-STEM) with electron energy loss spectroscopy (EELS) to enhance spatial resolution, along with computational simulations (such as DFT calculations) to predict electron deficiency impact on the electrocatalytic performance.

### 6.5 Utilizing unsaturated sites to couple hydrogen reduction with other important reactions

By employing various strategies, both electrophilic and nucleophilic active sites on MoS_2_ can be designed to meet the adsorption and activation requirements of different chemically active species. Introducing sulfur vacancies or other defects in the 2H phase MoS_2_ creates metallic-like sites with unpaired electrons, which effectively adsorb and activate electrophilic species such as CO_2_ and CO. Additionally, by doping high-electronegativity noble metals (such as Pt and Au), positively charged nucleophilic Mo sites (Mo^*δ*+^) can be engineered on the MoS_2_ surface to interact with electron-rich reactants. Under appropriate stoichiometric conditions, these active sites facilitate *in situ* hydrogenation reactions, yielding high-value products such as methanol, formic acid, or other hydrocarbons. This approach demonstrates that MoS_2_ not only exhibits excellent catalytic performance in HER but also holds promise for applications in hydrogenation, CO_2_ reduction reactions, and other significant processes.

## Data availability

No primary research results, software or code have been included and no new data were generated or analyzed as part of this review.

## Author contributions

All of the authors contributed to the manuscript preparation. Q. Z., X. R., and D. M. conceived the outline of the manuscript. Q. Z., and H. H. wrote the original draft of the manuscript. Z. C., X. R., and D. M. discussed and helped revise the manuscript.

## Conflicts of interest

The authors declare that they have no known competing financial interests or personal relationships that could have appeared to influence the work reported in this paper.

## References

[cit1] Wagner M., Meyer B., Setvin M., Schmid M., Diebold U. (2021). Nature.

[cit2] Huang W., He Q., Hu Y. P., Li Y. G. (2019). Angew. Chem., Int. Ed..

[cit3] Binninger T., Doublet M. L. (2022). Energy Environ. Sci..

[cit4] Hu K. L., Ohto T., Nagata Y., Wakisaka M., Aoki Y., Fujita J., Ito Y. (2021). Nat. Commun..

[cit5] Garrido-Barros P., Derosa J., Chalkley M. J., Peters J. C. (2022). Nature.

[cit6] Greeley J., Jaramillo T. F., Bonde J., Chorkendorff I. B., Nørskov J. K. (2006). Nat. Mater..

[cit7] Seh Z. W., Kibsgaard J., Dickens C. F., Chorkendorff I., Nørskov J. K., Jaramillo T. F. (2017). Science.

[cit8] Kim H. Y., Kim J. M., Ha Y., Woo J., Byun A., Shin T. J., Park K. H., Jeong H. Y., Kim H., Kim J. Y., Joo S. H. (2019). ACS Catal..

[cit9] Dudnik A. S., Weidner V. L., Motta A., Delferro M., Marks T. J. (2014). Nat. Chem..

[cit10] Zhou Q. Q., Zhou X. L., Zheng R. H., Liu Z. F., Wang J. D. (2022). Sci. Total Environ..

[cit11] Hinnemann B., Moses P. G., Bonde J., Jørgensen K. P., Nielsen J. H., Horch S., Chorkendorff I., Nørskov J. K. (2005). J. Am. Chem. Soc..

[cit12] Lukowski M. A., Daniel A. S., Meng F., Forticaux A., Li L., Jin S. (2013). J. Am. Chem. Soc..

[cit13] Fang M., Peng Y. Q., Wu P. W., Wang H., Xing L. X., Wang N., Tang C. M., Meng L., Zhou Y. K., Du L., Ye S. Y. (2024). Front. Energy.

[cit14] Jaramillo T. F., Jørgensen K. P., Bonde J., Nielsen J. H., Horch S., Chorkendorff I. (2007). Science.

[cit15] Jia Y. H., Zhang Y. C., Xu H. Q., Li J., Gao M., Yang X. T. (2024). ACS Catal..

[cit16] Meng H. S., Chen Z. J., Zhu J. L., You B., Ma T. Y., Wei W., Vernuccio S., Xu J., Ni B. J. (2024). Adv. Funct. Mater..

[cit17] Wang H. T., Lu Z. Y., Xu S. C., Kong D. S., Cha J. J., Zheng G. Y., Hsu P. C., Yan K., Bradshaw D., Prinz F. B., Cui Y. (2013). Proc. Natl. Acad. Sci. U.S.A..

[cit18] Tsai C., Li H., Park S., Park J., Han H. S., Nørskov J. K., Zheng X. L., Abild-Pedersen F. (2017). Nat. Commun..

[cit19] Wang H. T., Tsai C., Kong D. S., Chan K., Abild-Pedersen F., Nørskov J. K., Cui Y. (2015). Nano Res..

[cit20] Zhang J., Zhang Q. Y., Feng X. L. (2019). Adv. Mater..

[cit21] Fujisawa K., Carvalho B. R., Zhang T., Perea-Lopez N., Lin Z., Carozo V., Ramos S. L. L. M., Kahn E., Bolotsky A., Liu H., Elias A. L., Terrones M. (2021). ACS Nano.

[cit22] Voiry D., Fullon R., Yang J. U., Silva C. C. C., Kappera R., Bozkurt I., Kaplan D., Lagos M. J., Batson P. E., Gupta G., Mohite A. D., Dong L., Er D., Shenoy V. B., Asefa T., Chhowalla M. (2016). Nat. Mater..

[cit23] Park S., Kim C., Park S. O., Oh N. K., Kim U., Lee J., Seo J., Yang Y., Lim H. Y., Kwak S. K., Kim G., Park H. (2020). Adv. Mater..

[cit24] Chen Z. J., Yun S. N., Wu L., Zhang J. Q., Shi X. D., Wei W., Liu Y. W., Zheng R. J., Han N., Ni B. J. (2023). Nano-Micro Lett..

[cit25] Chen Z. J., Duan X. G., Wei W., Wang S. B., Ni B. J. (2020). Nano Energy.

[cit26] Yang W., Chen S. W. (2020). Chem. Eng. J..

[cit27] Wang Q. H., Kalantar-Zadeh K., Kis A., Coleman J. N., Strano M. S. (2012). Nat. Nanotechnol..

[cit28] Radisavljevic B., Radenovic A., Brivio J., Giacometti V., Kis A. (2011). Nat. Nanotechnol..

[cit29] Li Y., Duerloo K. A. N., Wauson K., Reed E. J. (2016). Nat. Commun..

[cit30] Wang H. M., Li C. H., Fang P. F., Zhang Z. L., Zhang J. Z. (2018). Chem. Soc. Rev..

[cit31] Lim Y. R., Han J. K., Yoon Y., Lee J. B., Jeon C., Choi M., Chang H., Park N., Kim J. H., Lee Z., Song W., Myung S., Lee S. S., An K. S., Ahn J. H., Lim J. S. (2019). Adv. Mater..

[cit32] Zhu D., Liu J., Zhao Y., Zheng Y., Qiao S. Z. (2019). Small.

[cit33] Yang H., Kim S., Chhowalla W. M., Lee Y. H. (2017). Nat. Phys..

[cit34] Chhowalla M., Shin H. S., Eda G., Li L.-J., Loh K. P., Zhang H. (2013). Nat. Chem..

[cit35] Khalil R. M. A., Hussain F., Rana A. M., Imran M., Murtaza G. (2019). Phys. E Low-dimens. Syst. Nanostruct..

[cit36] Yue Q., Kang J., Shao Z. Z., Zhang X. A., Chang S. L., Wang G., Qin S. Q., Li J. B. (2012). Phys. Lett. A.

[cit37] Huang Q. Z., Shen J. L., Lu Y., Ye R. D., Gong S. (2023). J. Phys. Chem. C.

[cit38] Zavala L. A., Kumar K., Martin V., Maillard F., Maugé F., Portier X., Oliviero L., Dubau L. (2023). ACS Catal..

[cit39] Yang F., Hu P., Yang F. F., Chen B., Yin F., Hao K., Sun R., Gao L., Sun Z., Wang K., Yin Z. (2023). Small.

[cit40] Cao Y. (2021). ACS Nano.

[cit41] Zhang Y. W., Wu Q., Seow J. Z. Y., Jia Y. J., Ren X., Xu Z. C. J. (2024). Chem. Soc. Rev..

[cit42] Linghu Y. Y., Li N., Du Y. P., Wu C. (2019). Phys. Chem. Chem. Phys..

[cit43] Xu J., Shao G. L., Tang X., Lv F., Xiang H. Y., Jing C. F., Liu S., Dai S., Li Y. G., Luo J., Zhou Z. (2022). Nat. Commun..

[cit44] Huang Y. C., Sun Y. H., Zheng X. L., Aoki T., Pattengale B., Huang J., He X., Bian W., Younan S., Williams N., Hu J., Ge J. X., Pu N., Yan X. X., Pan X. Q., Zhang L. J., Wei Y. G., Gu J. (2019). Nat. Commun..

[cit45] Zhang L. Y., Li M., Zou A. Q., Yu S. H., Xiong T., Wang L., He J. J., Fu Q., Sun K., Chua D. H. C., Xue J. M. (2019). ACS Appl. Energy Mater..

[cit46] Wang X., Zhang Y. W., Si H. N., Zhang Q. H., Wu J., Gao L., Wei X. F., Sun Y., Liao Q. L., Zhang Z., Ammarah K., Gu L., Kang Z., Zhang Y. (2020). J. Am. Chem. Soc..

[cit47] Fang Y. Q., Hu X. Z., Zhao W., Pan J., Wang D., Bu K. J., Mao Y. L., Chu S. F., Liu P., Zhai T. Y., Huang F. Q. (2019). J. Am. Chem. Soc..

[cit48] Zhang T., Liu Y. P., Yu J., Ye Q. T., Yang L., Li Y., Fan H. J. (2022). Adv. Mater..

[cit49] Shao G. L., Yang M. Q., Xiang H. Y., Luo S., Xue X. X., Li H. M., Zhang X., Liu S., Zhou Z. (2023). Nano Res..

[cit50] Zhang T., Ye Q. T., Han Z. Y., Liu Q. Y., Liu Y. P., Wu D. S., Fan H. J. (2024). Nat. Commun..

[cit51] Wang J. W., He L. Q., Zhang Y. H., Nong H. Y., Li S. N., Wu Q. K., Tan J. Y., Liu B. L. (2024). Adv. Mater..

[cit52] Klimov V. I., Mikhailovsky A. A., McBranch D. W., Leatherdale C. A., Bawendi M. G. (2000). Phys. Rev. B:Condens. Matter Mater. Phys..

[cit53] Wang F., Shifa T. A., Zhan X., Huang Y., Liu K., Cheng Z., Jiang C., He J. (2015). Nanoscale.

[cit54] Novoselov K. S., Jiang D., Schedin F., Booth T. J., Khotkevich V. V., Morozov S. V., Geim A. K. (2005). Proc. Natl. Acad. Sci. U.S.A..

[cit55] Ji Q. Q., Li C., Wang J. L., Niu J. J., Gong Y., Zhang Z. P., Fang Q. Y., Zhang Y., Shi J. P., Liao L., Wu X. S., Gu L., Liu Z. F., Zhang Y. F. (2017). Nano Lett..

[cit56] Zhang Z. P., Niu J. J., Yang P. F., Gong Y., Ji Q. Q., Shi J. P., Fang Q. Y., Jiang S. L., Li H., Zhou X. B., Gu L., Wu X. S., Zhang Y. F. (2017). Adv. Mater..

[cit57] Zhang Y., Zhang Y. F., Ji Q. Q., Ju J., Yuan H. T., Shi J. P., Gao T., Ma D. L., Liu M. X., Chen Y. B., Song X. J., Hwang H. Y., Cui Y., Liu Z. F. (2013). ACS Nano.

[cit58] Song J. G., Park J. S., Lee W., Choi T., Jung H., Lee C. W., Hwang S. H., Myoung J. M., Jung J. H., Kim S. H., Matras C. L., Kim H. (2014). ACS Nano.

[cit59] Shi J. P., Ma D. L., Han G. F., Zhang Y., Ji Q. Q., Gao T., Sun J. Y., Song X. J., Li C., Zhang Y. S., Lang X. Y., Zhang Y. F., Li Z. F. (2014). ACS Nano.

[cit60] Shi J. P., Wang X. N., Zhang S., Xiao L. F., Huan Y. H., Gong Y., Zhang Z. P., Li Y. C., Zhou X. B., Hong M., Fang Q. Y., Zhang Q., Liu X. F., Gu L., Liu Z. F., Zhang Y. F. (2017). Nat. Commun..

[cit61] Chang K., Hai X., Pang H., Zhang H. B., Shi L., Liu G. G., Liu H. M., Zhao G. X., Li M., Ye J. H. (2016). Adv. Mater..

[cit62] Zhang L. J., Zunger A. (2015). Nano Lett..

[cit63] Kibsgaard J., Chen Z., Reinecke B. N., Jaramillo T. F. (2012). Nat. Mater..

[cit64] Huang Z. Y., Qi X., Yang H., He C. Y., Wei X. L., Peng X. Y., Zhong J. X. (2015). J. Phys. D.

[cit65] Kong D. S., Wang H. T., Cha J. J., Pasta M., Koski K. J., Yao J., Cui Y. (2013). Nano Lett..

[cit66] Geng X., Wu W., Li N., Sun W., Armstrong J., Al-hilo A., Brozak M., Cui J., Chen T. P. (2014). Adv. Funct. Mater..

[cit67] Yang Y., Fei H., Ruan G., Xiang C., Tour J. M. (2014). Adv. Mater..

[cit68] Deng J., Li H. B., Wang S. H., Ding D., Chen M. S., Liu C., Tian Z. Q., Novoselov K. S., Ma C., Deng D. H., Bao X. H. (2017). Nat. Commun..

[cit69] Jiang Y. M., Li X., Yu S. J., Jia L. P., Zhao X. J., Wang C. M. (2015). Adv. Funct. Mater..

[cit70] Ren X. P., Pang L. Q., Zhang Y. X., Ren X. D., Fan H. B., Liu S. Z. (2015). J. Mater. Chem. A.

[cit71] Nayak A. P., Pandey T., Voiry D., Liu J., Moran S. T., Sharma A., Tan C., Chen C. H., Li L. J., Chhowalla M., Lin J. F., Singh A. K., Akinwande D. (2015). Nano Lett..

[cit72] Nayak A. P., Bhattacharyya S., Zhu J., Liu J., Wu X., Pandey T., Jin C. Q., Singh A. K., Akinwande D., Lin J. F. (2014). Nat. Commun..

[cit73] Zhao Z., Zhang H. J., Yuan H. T., Wang S. B., Lin Y., Zeng Q. S., Xu G., Liu Z. X., Solanki G. K., Patel K. D., Cui Y., Hwang H. Y., Mao W. L. (2015). Nat. Commun..

[cit74] Yu H. Y., Liu G. B., Tang J. J., Xu X. D., Yao W. (2017). Sci. Adv..

[cit75] Lin Y. C., Dumcenco D. O., Huang Y. S., Suenaga K. (2014). Nat. Nanotechnol..

[cit76] Katagiri Y., Nakamura T., Ishii A., Ohata C., Hasegawa M., Katsumoto S., Cusati T., Fortunelli A., Iannaccone G., Fiori G. (2016). Nano Lett..

[cit77] Guo X. W., Song E. H., Zhao W., Xu S. M., Zhao W. L., Lei Y. J., Fang Y. Q., Liu J. J., Huang F. Q. (2022). Nat. Commun..

[cit78] Wu W. Z., Niu C. Y., Wei C., Jia P. Y., Li C., Xu P. Q. (2019). Angew. Chem., Int. Ed..

[cit79] Feng C. Y., Wu Z. P., Huang K. W., Ye J. H., Zhang H. B. (2022). Adv. Mater..

[cit80] Xu J., Xue X. X., Shao G. L., Jing C. F., Dai S., He K., Jia P. P., Wang S., Yuan Y. F., Luo J., Lu J. (2023). Nat. Commun..

[cit81] Zhou Y., Zhang J., Song E. H., Lin J. H., Zhou J. D., Suenaga K., Zhou W., Liu Z., Liu J. J., Lou J., Fan H. J. (2020). Nat. Commun..

[cit82] Luo Z. Y., Ouyang Y. X., Zhang H., Xiao M. L., Ge J. J., Jiang Z., Wang J. L., Tang D. M., Cao X. Z., Liu C. P., Xing W. (2018). Nat. Commun..

[cit83] Liu Y. G., Guan S. J., Du X. S., Chen Y. R., Yang Y., Chen K. L., Zheng Z. W., Wang X., Shen X. Q., Hu C. L., Li X. B. (2023). Energy Fuels.

[cit84] Zhang J. M., Xu X. P., Yang L., Cheng D. J., Cao D. P. (2019). Small Methods.

[cit85] Liu B., Cheng Y., Cao B., Hu M., Jing P., Gao R., Du Y., Zhang J., Liu J. (2021). Appl. Catal., B.

[cit86] Zang Y. P., Niu S. W., Wu Y. S., Zheng X. S., Cai J. Y., Ye J., Xie Y. F., Liu Y., Zhou J. B., Zhu J. F., Liu X. J., Wang G. M., Qian Y. (2019). Nat. Commun..

[cit87] Yang Q., Wang Z., Dong L., Zhao W., Jin Y., Fang L., Hu B., Dong M. (2019). J. Phys. Chem. C.

[cit88] Ge J., Zhang D., Jin J., Han X., Wang Y., Zhang F., Lei X. (2021). Mater. Today Energy.

[cit89] Shi Y., Zhang D., Miao H., Wu X., Wang Z., Zhan T., Lai J., Wang L. (2022). Sci. China: Chem..

[cit90] Jiang L., Zhang Y. J., Luo X. H., Yu L., Li H. X., Li Y. J. (2021). Chem. Eng. J..

[cit91] Zheng Z. L., Yu L., Gao M., Chen X. Y., Zhou W., Ma C., Wu L. H., Zhu J. F., Meng X. Y., Hu J. T., Tu Y. C., Wu S. S., Mao J., Tian Z. Q., Deng D. H. (2020). Nat. Commun..

[cit92] Li H., Yu F., Ling X., Wan H., Zhang M., Zhou Y., Wei J., Lu F., Zhang X., Zeng X., Zhou M. (2021). Nanotechnology.

[cit93] Han D., Luo Z., Li Y., Gao N., Ge J., Liu C., Xing W. (2020). Appl. Surf. Sci..

[cit94] Luo Z. Y., Zhang H., Yang Y. Q., Wang X., Li Y., Jin Z., Jiang Z., Liu C. P., Xing W., Ge J. J. (2020). Nat. Commun..

[cit95] Luo Y. T., Zhang S. Q., Pan H. Y., Xiao S. J., Guo Z. L., Tang L., Khan U., Ding B. F., Li M., Cai Z. Y., Zhao Y., Lv W., Feng Q. L., Zou X. L., Lin J. H., Cheng H. M., Liu B. L. (2020). ACS Nano.

[cit96] Wang J., Cheng C., Yuan Q., Yang H., Meng F. Q., Zhang Q. H., Gu L., Cao J. L., Li L. G., Haw S. C., Shao Q., Zhang L., Cheng T., Jiao F., Huang X. Q. (2022). Chem.

[cit97] Yang J., Cao Y. F., Zhang S. Y., Shi Q. W., Chen S. Y., Zhu S. C., Li Y. S., Huang J. F. (2023). Small.

[cit98] Pham V. P., Yeom G. Y. (2016). Adv. Mater..

[cit99] Zhou Q. Q., Wang Z. Y., Yuan H. D., Wang J. D., Hu H. (2023). Appl. Catal., B.

[cit100] Shi Z. Y., Zhang X., Lin X. Q., Liu G. G., Ling C. Y., Xi S. B., Chen B., Ge Y. Y., Tan C. L., Lai Z. C., Huang Z. Q., Ruan X. Y., Zhai L., Li L. J., Li Z. J., Wang X. X., Nam G. H., Liu J. W., He Q. Y., Guan Z. Q., Wang J. L., Lee C. S., Kucernak A. R. J., Zhang H. (2023). Nature.

[cit101] Qi K., Cui X. Q., Gu L., Yu S. S., Fan X. F., Luo M. C., Xu S., Li N. B., Zheng L. R., Zhang Q. H., Ma J. Y., Gong Y., Lv F., Wang K., Huang H. H., Zhang W., Guo S. J., Zheng W. T., Liu P. (2019). Nat. Commun..

[cit102] Hu H., Zhou Q. Q., Wang Z. Y., Wang J. D., Chen Y. M., Han Y. X. (2024). Chin. Sci. Bull..

[cit103] Xiong H., Du C. F., Ma Z. L., Zhi R. C., Hao S. S., Zhao X. Y., Liu Z., Xu F., Wang H. Q. (2024). Adv. Funct. Mater..

[cit104] Chen W. S., Gu J. J., Du Y. P., Song F., Bu F. X., Li J. H., Yuan Y., Luo R. C., Liu Q. L., Zhang D. (2020). Adv. Funct. Mater..

[cit105] Sun Z. M., Lin L., Yuan M. W., Yao H. Y., Deng Y. J., Huang B. H., Li H. F., Sun G. B., Zhu J. (2022). Nano Energy.

[cit106] Hong Z. A., Hong W. T., Wang B. C., Cai Q., He X., Liu W. (2023). Chem. Eng. J..

[cit107] Liu M. Q., Wang J. A., Klysubun W., Wang G. G., Sattayaporn S., Li F., Cai Y. W., Zhang F. C., Yu J., Yang Y. (2021). Nat. Commun..

[cit108] Pi C. R., Li X. X., Zhang X. M., Song H., Zheng Y., Gao B., Kızılaslan A., Chu P. K., Huo K. F. (2022). Small.

[cit109] Xue Y. H., Zhang Q., Wang W. J., Cao H., Yang Q. H., Fu L. (2017). Adv. Energy Mater..

[cit110] Wu Y. C., Li D. f., Wu C. L., Hwang H. Y., Cui Y. (2023). Nat. Rev. Mater..

[cit111] Xu D., Zhang S. N., Chen J. S., Li X. H. (2023). Chem. Rev..

[cit112] Chen Z. J., Ma T. Y., Wei W., Wong W. Y., Zhao C., Ni B. J. (2024). Adv. Mater..

[cit113] Liu C., Zou X. M., Lv Y. W., Liu X. Q., Ma C., Li K. L., Liu Y., Chai Y., Liao L., He J. (2024). Nat. Nanotechnol..

[cit114] Yuan H. T., Wang H. T., Cui Y. (2015). Acc. Chem. Res..

[cit115] Huang X., Zeng Z. Y., Zhang H. (2013). Chem. Soc. Rev..

[cit116] Chen Z. X., Leng K., Zhao X. X., Malkhandi S., Tang W., Tian B. B., Dong L., Zheng L. R., Lin M., Yeo B. S., Loh K. P. (2017). Nat. Commun..

[cit117] Zhao X. X., Song P., Wang C. C., Riis-Jensen A. C., Fu W., Deng Y., Wan D. Y., Kang L. X., Ning S. C., Dan J. D., Venkatesan T., Liu Z., Zhou W., Thygesen K. S., Luo X., Pennycook S. J., Loh K. P. (2020). Nature.

[cit118] Tsai C., Pedersen F. A., Nørskov J. K. (2014). Nano Lett..

[cit119] Kang J. H., Liu W., Sarkar D., Jena D., Banerjee K. (2014). Phys. Rev. X.

[cit120] Li W. S., Gong X. S., Yu Z. H., Ma L., Sun W. J., Gao S., Köroğlu Ç., Wang W. F., Liu L., Li T. T., Ning H. K., Fan D. X., Xu Y. F., Tu X. C., Xu T., Sun L. T., Wang W. H., Lu J. P., Ni Z. H., Li J., Duan X. D., Wang P., Nie Y. F., Qiu H., Wang X. R. (2023). Nature.

[cit121] Bae C., Ho T. A., Kim H., Lee S., Lim S., Kim M., Yoo H., Montero-Moreno J. M., Park J. H., Shin H. (2017). Sci. Adv..

[cit122] Liu H. M., Xie R. K., Luo Y. T., Cui Z. C., Yu Q. M., Gao Z. Q., Zhang Z. Y., Yang F. N., Kang X., Ge S. Y., Li S. H., Gao X. F., Chai G. L., Liu L., Liu B. L. (2022). Nat. Commun..

[cit123] Yu Q. M., Zhang Z. Y., Qiu S. Y., Luo Y. T., Liu Z. B., Yang F. N., Liu H. M., Ge S. Y., Zou X. L., Ding B. F., Ren W. C., Cheng H. M., Sun C. H., Liu B. L. (2021). Nat. Commun..

[cit124] Cheng M., Cao N., Wang Z., Wang K., Pu T. C., Li Y. K., Sun T. L., Yue X. Y., Ni W. K., Dai W. X., He Y., Shi Y., Zhang P., Zhu Y. H., Xie P. F. (2024). ACS Nano.

[cit125] Ma T., Cao H., Li S., Cao S. J., Zhao Z. Y., Wu Z. H., Yan R., Yang C. D., Wang Y., van Aken P. A., Qiu L., Wang Y. G., Cheng C. (2022). Adv. Mater..

[cit126] Liu L. N., Wu J. X., Wu L. Y., Ye M., Liu X. Z., Wang Q., Hou S. Y., Lu P. F., Sun L. F., Zheng J. Y., Xing L., Gu L., Jiang X. W., Xie L. M., Jiao L. Y. (2018). Nat. Mater..

[cit127] Benson E. E., Zhang H. Y., Schuman S. A., Nanayakkara S. U., Bronstein N. D., Ferrere S., Blackburn J. L., Miller E. M. (2018). J. Am. Chem. Soc..

[cit128] Kwon I. S., Kwak I. H., Debela T. T., Abbas H. G., Park Y. C., Ahn J. P., Park J., Kang H. S. (2020). ACS Nano.

[cit129] Wu H. B., Xia B. Y., Yu L., Yu X. Y., Lou X. W. (2015). Nat. Commun..

[cit130] Liu H. J., He Q., Jiang H. L., Lin Y. X., Zhang Y. K., Habi M., Chen S. G., Song L. (2017). ACS Nano.

[cit131] Chen G. F., Ma T. Y., Liu Z. Q., Li N., Su Y. Z., Davey K., Qiao S. Z. (2016). Adv. Funct. Mater..

[cit132] Xue Y. Q., Bai X. J., Xu Y. Y., Yan Q., Zhu M., Zhu K., Ye K., Yan J., Cao D. X., Wang G. L. (2021). Composites, Part B.

[cit133] An C. H., Kang W., Deng Q. B., Hu N. (2022). Rare Met..

